# Targeting CSF1R in myeloid-derived suppressor cells: insights into its immunomodulatory functions in colorectal cancer and therapeutic implications

**DOI:** 10.1186/s12951-024-02584-4

**Published:** 2024-07-11

**Authors:** Xin Tong, Shifeng Qiao, Zhe Dong, Xiaohui Zhao, Xiaxia Du, Wei Niu

**Affiliations:** 1 Department of General Surgery, The First Affiliated Hospital of Jinzhou Medical University, Jinzhou, 121000 P. R. China; 2 Department of Medical Oncology, The First Affiliated Hospital of Jinzhou Medical University, Jinzhou, 121000 P. R. China; 3 Department of Rehabilitation, The Third Affiliated Hospital of Jinzhou Medical University, Jinzhou, 121000 P. R. China; 4 Department of Gastroenterology, The First Affiliated Hospital of Jinzhou Medical University, No. 2, Section 5, Renmin Street, Guta District, Jinzhou, Liaoning Province 121000 P. R. China

**Keywords:** Colorectal cancer, Myeloid-derived suppressor cells, CSF1R, JAK/STAT3, LNCs@CSF1R siRNA, Anti-PD-1, Immune suppression, Fatty acid metabolism

## Abstract

**Objective:**

This study aimed to investigate the critical role of MDSCs in CRC immune suppression, focusing on the CSF1R and JAK/STAT3 signaling axis. Additionally, it assessed the therapeutic efficacy of LNCs@CSF1R siRNA and anti-PD-1 in combination.

**Methods:**

Single-cell transcriptome sequencing data from CRC and adjacent normal tissues identified MDSC-related differentially expressed genes. RNA-seq analysis comprehensively profiled MDSC gene expression in murine CRC tumors. LNCs@CSF1R siRNA nanocarriers effectively targeted and inhibited CSF1R. Flow cytometry quantified changes in MDSC surface markers post-CSF1R inhibition. RNA-seq and pathway enrichment analyses revealed the impact of CSF1R on MDSC metabolism and signaling. The effect of CSF1R inhibition on the JAK/STAT3 signaling axis was validated using Colivelin and metabolic assessments. Glucose and fatty acid uptake were measured via fluorescence-based flow cytometry. The efficacy of LNCs@CSF1R siRNA and anti-PD-1, alone and in combination, was evaluated in a murine CRC model with extensive tumor section analyses.

**Results:**

CSF1R played a significant role in MDSC-mediated immune suppression. LNCs@CSF1R siRNA nanocarriers effectively targeted MDSCs and inhibited CSF1R. CSF1R regulated MDSC fatty acid metabolism and immune suppression through the JAK/STAT3 signaling axis. Inhibition of CSF1R reduced STAT3 activation and target gene expression, which was rescued by Colivelin. Combined treatment with LNCs@CSF1R siRNA and anti-PD-1 significantly slowed tumor growth and reduced MDSC abundance within CRC tumors.

**Conclusion:**

CSF1R via the JAK/STAT3 axis critically regulates MDSCs, particularly in fatty acid metabolism and immune suppression. Combined therapy with LNCs@CSF1R siRNA and anti-PD-1 enhances therapeutic efficacy in a murine CRC model, providing a strong foundation for future clinical applications.

**Supplementary Information:**

The online version contains supplementary material available at 10.1186/s12951-024-02584-4.

## Introduction

Colorectal cancer remains one of the leading causes of cancer-related deaths globally, posing a significant burden to healthcare systems [1]. Its prevalence, coupled with the devastating impact it can have on patients and families, has led to extensive research efforts to identify effective treatment modalities [2, 3]. Over recent years, the emergence of immunotherapy has shown tremendous promise in enhancing the treatment landscape for colorectal cancer patients [4].

However, the intricate and multifaceted tumor microenvironment (TME) often complicates the clinical efficacy of immunotherapeutic approaches. Within the TME, a multitude of immune cells coexist, some of which may be harnessed for therapeutic benefits, while others might play roles that counteract the therapeutic interventions [5]. One such cellular population, the myeloid-derived suppressor cells (MDSCs), has been the subject of significant attention owing to its robust immunosuppressive capabilities [6, 7]. MDSCs not only dampen the body’s natural anti-tumor immune response but also create an environment conducive to tumor growth and progression [8]. This has prompted researchers to delve deeper into understanding the underlying mechanisms governing MDSC function and regulation in hopes of countering their suppressive actions and thereby enhancing immunotherapeutic outcomes [9].

Central to the regulatory machinery of MDSCs is the colony-stimulating factor 1 receptor (CSF1R). Accumulating evidence has highlighted the pivotal role of CSF1R in various tumor contexts, underscoring its influence on the proliferation, differentiation, and most importantly, the immunosuppressive function of MDSCs [10, 11]. Activation of MDSCs through CSF1R-mediated pathways strengthens their inhibitory actions on immune effector cells, notably the CD8 + T cells, which are essential for anti-tumor immunity [12, 13]. The consequence of this heightened suppression is a tumor environment that can resist immune-mediated destruction, diminishing the potential benefits of immunotherapies [14].

Further complicating the picture is the JAK/STAT3 signaling pathway. This pathway, renowned for its role in several malignancies, including colon cancer, encompasses a wide range of cellular processes from cell survival and migration to proliferation [15, 16]. Of note, there’s emerging evidence suggesting a synergy between CSF1R signaling and the JAK/STAT3 axis, whereby their coordinated action bolsters the immunosuppressive capacities of MDSCs, further driving tumor immune evasion [6, 17, 18].

In light of these insights, there’s a compelling rationale to target the CSF1R and JAK/STAT3 interplay as a means to modulate MDSC function, especially in the realm of fatty acid metabolism—a pivotal aspect of MDSC biology. This research aims to elucidate the intricate relationship between CSF1R and MDSC lipid metabolism, with an overarching goal of appraising the potential therapeutic benefits of a novel nanocarrier, LNCs@CSF1R siRNA, in tandem with anti-PD-1 antibodies for the treatment of colorectal cancer in pre-clinical models.

## Materials and methods

### Ethical statement

The experimental procedures involving animal for this study received approval from the First Affiliated Hospital of Jinzhou Medical University. The animals were cared and handled in accordance with the Guide for the Care and Use of Laboratory Animals.

### Download scRNA-seq data

Retrieve the scRNA-seq dataset GSE200997 related to human colon cancer from the Gene Expression Omnibus (GEO) database, accessible at https://www.ncbi.nlm.nih.gov/geo/. The study has the accession number GSE200997, and the sequencing platform used was GPL21697. It consists of 16 samples of human colon cancer tissue (GSM6048346, GSM6048347, GSM6048348, GSM6048349, GSM6048350, GSM6048351, GSM6048352, GSM6048353, GSM6048354, GSM6048355, GSM6048356, GSM6048357, GSM6048358, GSM6048359, GSM6048360, GSM6048361), as well as 7 samples of adjacent normal tissue (GSM6048362, GSM6048363, GSM6048364, GSM6048365, GSM6048366, GSM6048367, GSM6048368). Because the GEO database provides access to public data, ethical approval or informed consent is not necessary for this part of the study [19].

### Single-cell sequencing analysis

We utilized the Seurat package (version 3.1) in R software to perform standard downstream analysis on scRNA-seq data. Specifically, we filtered out cells with fewer than 200 detected genes and genes detected in less than 3 cells. Additionally, we controlled the mitochondrial proportion to be below 10%. Afterwards, use the LogNormalize method to normalize the data [20].

Next, the FindClusters function should cluster and visualize the cells using the RunUMAP function. Identifying specific marker genes for cell clusters was accomplished using the FindMarkers function in the Seurat package. When identifying differentially expressed genes (DEGs) within a specific cluster, the Wilcoxon rank-sum test compares the cells within that cluster to all others. The critical value for identifying differentially expressed genes (DEGs) with statistical significance is a Bonferroni-adjusted p-value less than 0.05. Identify known marker genes specific to each cell lineage and utilize the online website CellMarker for cell annotation [21]. Meanwhile, we analyzed cell communication using the CellChat package in the R language.

### Differential gene expression screening

To filter differentially expressed genes (DEGs), the limma package in R software could be utilized. The differentially expressed genes (DEGs) of MDSCs in both Normal and Tumor samples were filtered based on the criteria of |logFC| > 0.5 and P.adjust < 0.05. To generate a volcano plot of differentially expressed genes (DEGs), the pheatmap package in the R software is utilized [22].

### GO and KEGG enrichment analysis

We utilized the clusterProfiler, org.Hs.eg.db, enrichplot, and ggplot2 packages in R to conduct Gene Ontology (GO) and KEGG enrichment analysis on the identified DEGs. Bubble plots were generated to visualize the enrichment results for three Gene Ontology (GO) categories: biological process (BP), cellular component (CC), and molecular function (MF). Furthermore, a separate bubble plot was created for the KEGG enrichment analysis results [23].

### Preparation and characterization of LNCs@CSF1R siRNA

The materials and reagents utilized in this study consisted of 1,2-distearoyl-sn-glycero-3-phosphoethanolamine-N-amino-2000 (DSPE-PEG2000) and DSPE-PEG2000-mal-eimide (DSPE-PEG-Mal), both obtained from Avanti Polar Lipids (Alabaster, AL, USA). Stearic acid (SA) and polyethyleneimine (PEI 1800) were purchased from Aladin (Shanghai, China). EDC (1-(3-(dimethylamino) propyl)-3-ethylcarbodiimide hydrochloride) and NHS (N-Hydroxy-succinimide) were obtained from J&K Scientific (Beijing, China). The CSF1R siRNA (5’-CAGGCAGTACCACCATCCACTTGTA-3’) was synthesized by GenePharma, located in Shanghai, China.

Stearic acid (SA, 142.4 mg) should be dissolved in 10 mL of dimethyl sulfoxide. Subsequently, EDCI (115 mg) and NHS (69 mg) should be added. The mixture should be stirred at room temperature for 2 h to ensure the complete activation of the carboxyl groups in stearic acid. Subsequently, 1504.2 mg of polyethyleneimine (PEI 1800, Aladin) should be added. After being stirred and cultured for 24 h, the remaining stearic acid should be extracted using ethyl acetate. The obtained PSA is then purified through dialysis in a bag containing distilled water for 48 h. Subsequently, the dialysate undergoes freeze-drying to obtain the final product known as PSA [24].

The conjugation of Anti-Gr-1 (ab25377, Abcam, UK) with DSPE-PEG2000-Mal was carried out using a Michael addition reaction. Under the catalysis of a tertiary amine, DSPE-PEG2000-Mal and Anti-Gr-1 were dissolved in a mixture of chloroform and methanol (2:1) at a molar ratio of 1:1.5. The mixture should be incubated at room temperature in the presence of argon gas for 48 h. The reaction is complete once DSPE-PEG2000-Mal is no longer detected on thin-layer chromatography (TLC). Next, the mixture should be dialyzed in a dialysis bag using distilled water. The product should be stored below 20 °C after freeze-drying for future use.

DSPE-PEG, DSPEPEG-Anti-Gr-1, and PSA were dissolved in methanol at a mass ratio of 8:2:1 (w/w) and transferred into a round-bottom flask. Organic solvents could be evaporated, and a lipid membrane could be formed using a rotary evaporator (RE-2000 A, manufactured in China by Shanghai Yarong). The sample was hydrated with PBS buffer (pH 7.4) at 37 °C for 20 min. Subsequently, a probe sonicator (SCIENTZ18-A, China, Ningbo Xinzhi Bio-Tech Co., Ltd.) was used to sonicate the mixture at 80 W for 2 min, forming micelles. The CSF1R siRNA should be diluted in nucleic acid enzyme-free water and then mixed with various micelles at a weight ratio of 1:5. After centrifuging for 15 s, incubate at room temperature for 15 min to allow the formation of a complex (LNCs@CSF1R siRNA) (Figure S1A).

The LNCs@CSF1R siRNA average diameter, as determined by dynamic light scattering (DLS), is 82.6 ± 3.6 nm (Figure S1B). The Zeta potential is -42.31 ± 5.89 mV (Figure S1C). Transmission electron microscopy (TEM; LIBRA120, Zeiss, https://zeiss.com.cn/) revealed that the nanospheres displayed a spherical shape and a uniform size distribution (Figure S1D). The inclusion of DSPE-PEG2000 enhances the colloidal stability of liposomes, enabling them to retain their size in phosphate-buffered saline (PBS) containing 10% fetal bovine serum (FBS) at 37 °C for 72 h without any alterations in size (Figure S1E). This result indicates that LNCs@CSF1R siRNA exhibits excellent stability under typical physiological conditions [25].

### A mouse model of colon cancer was induced using AOM (azoxymethane) and DSS (sodium dextran sulfate)

The control group in the AOM and DSS-induced mouse model experiments of colon cancer consisted of untreated C57BL/6 mice (219, Beijing Vitonlihua Experimental Animal Co., Ltd.) aged 6–8 weeks, weighing approximately 18–22 g. Mice were intraperitoneally injected with a single dose of the genotoxic colon carcinogen AOM (12.5 mg/kg body weight, A5486, Sigma). On the 7th day, 1.5% DSS (60316EG25, Yisheng Bio-Technology Co., Ltd., Shanghai) should be administered in drinking water continuously for 7 days, followed by a switch to normal drinking water for 14 days. The loop runs for three iterations (Figure S2).

If the weight of the mice decreases by more than 20%, a fecal occult blood test is conducted, and a total score of 5 is assigned. The scoring criteria range from no occult blood (0 points) to severe occult blood (4–5 points) and may vary. Mice with a score of 3 or higher will be identified as models for colon cancer [26]. Following successful confirmation of the colon cancer mouse model, LNCs@CSF1R siRNA was administered via the tail vein at a dose of 8 mg/kg (based on body weight), with injections given every other day for a total of 5 times [27].

The control group received an injection of PBS. After 28 days, the mice were euthanized using intraperitoneal injection of pentobarbital sodium (100 mg/kg), and appropriate samples were collected for transcriptome sequencing. Each group should utilize a minimum of 6 mice for the succeeding experiments.

### Separation and culture of MDSCs

Femurs from C57BL/6 mice were collected and rinsed in a medium containing 10% FBS RPMI-1640 (R8758, Sigma) to obtain bone marrow precursor cells. These cells were then cultured in a modified DMEM medium (11965092, Thermo, USA) at 2 × 10^5^ cells/mL density. The culture medium was supplemented with 10% fetal bovine serum (10099141 C, Thermo, USA), 100 U/mL penicillin, 100 mg/mL streptomycin (10378016, Thermo, USA), and 1.5 mM L-glutamine (21051040, Thermo, USA). To generate myeloid-derived suppressor cells (MDSCs) during the cultivation process, 100 ng/mL of mouse G-CSF (Catalog #: 414-CS, R&D Systems), 250 U/mL of mouse GM-CSF (Catalog #: 415-ML, R&D Systems), and 80 ng/mL of IL-13 (Catalog #: 413-ML, R&D Systems) should be added [28].

To isolate MDSCs derived from mouse tumors of colon cancer origin, the tumors were enzymatically digested with DNase (18047019, Thermo, USA) and Liberase (5401119001, Roche, Switzerland) at 37 °C for 1 h. CD11b + Gr-1 + MDSCs were subsequently isolated from the single-cell suspension of the tumors using flow cytometry [29].

To treat MDSC, we added 20 µg/mL of LNCs@CSF1R siRNA and 50 µg/mL of Colivelin (HY-P1061, MedChemExpress) separately for 24 h and 4 h, respectively.

### Flow cytometry

The antibodies used to identify cell subsets included the following: anti-CD11b (clone: M1/70), anti-Gr1 (clone: RB6-8C5), anti-Ly6C (clone: AL-21), anti-Ly6G (clone: 1A8), anti-CD8 (clone: 53 − 6.7), anti-IFN-γ (clone: 2E2), anti-GzmB (clone: GB11), anti-CD206 (clone: Y17-505), anti-F4/80 (clone: T45-2342), and anti-TNF-α (clone: MP6-XT22) provided by BD Biosciences. The analysis used a flow cytometer (BD Biosciences, BD LSRFortessa, USA) [29].

### Immunofluorescence detection of CD11b + Gr-1 + MDSC

To remove the wax, slice the mouse tumor tissues embedded in paraffin using xylene. Afterward, rehydrate the tissues with a series of gradient alcohols. Next, antigen retrieval was conducted using a 0.01 mol/L sodium citrate buffer (pH 6.0). The slides were blocked with 1% BSA containing 0.1% Triton X-100 in PBS for 60 min. Then, the slides were incubated overnight at 4℃ with Alexa Fluor® 488 fluorescent Anti-CD11b antibody (ab307387, 1:50, Abcam) and Anti-Gr-1 (ab25377, 1:20, Abcam). Subsequently, wash the sample three times with phosphate-buffered saline (PBS) and incubate it with a goat anti-rat IgG H&L secondary antibody (ab150160, 1:1000, Abcam) conjugated with Alexa Fluor® 594. This incubation should be performed in the absence of light for 2 h. Remove any excess secondary antibody by washing with PBS, and treat the sample with DAPI (1:1000, #8961, Cell Signaling) for 1 h, avoiding exposure to light. Following three additional washes with PBS, the sample could be observed under a confocal microscope (Leica, STELLARIS 5, Germany) [29].

### High-throughput transcriptome sequencing

Sequencing samples were obtained by isolating CD11b + Gr-1 + myeloid-derived suppressor cells (MDSCs) from the bone marrow of C57BL/6 mice (*n* = 3), tumors of mice with colon cancer (*n* = 3), colon cancer mice injected with PBS (*n* = 3), and colon cancer mice injected with LNCs@CSF1R siRNA (*n* = 3).

In the process of high-throughput transcriptome sequencing, the following specific operation was conducted: Total RNA was extracted from the mentioned MDSC using Trizol reagent (Thermo, 16096020, USA). RNA concentration, purity, and integrity were assessed using the Qubit® 2.0 Fluorometer® (Thermo, Q33238, USA) with the Qubit® RNA Analysis Kit (Shanghai Baoji Biotechnology Co., Ltd, HKR2106-01, Shanghai, China), the NanoDrop spectrophotometer (IMPLEN, USA), and the RNA Nano 6000 Analysis Kit (Agilent, 5067 − 1511, USA) on the Bioanalyzer 2100 system. The RNA concentration was higher than 100 ng/µL, and the purity was determined based on the 260/280 ratio, which ranged between 1.8 and 2.1. Each sample has a total RNA content of 3 µg, which serves as input material for RNA sample preparation. To generate the cDNA library, follow the manufacturer’s recommendations and use the NEBNext® UltraTM RNA Library Prep Kit (NEB, E7435L, Beijing, China) designed for Illumina® (USA). Assess the quality of the library using an Agilent Bioanalyzer 2100 system. Per the manufacturer’s instructions, the indexed-encoded samples were clustered using the TruSeq PE Cluster Kit v3 cBot HS (PE-401-3001, Illumina, USA) on the cBot Cluster Generation System. Following cluster generation, library preparation was conducted on the Illumina HiSeq 550 platform, producing 125 bp/150 bp paired-end reads [30, 31].

FastQC software version 0.11.8 was employed to evaluate the quality of paired-end reads derived from raw sequencing data. The raw data was preprocessed using Cutadapt software version 1.18 to eliminate Illumina sequencing adaptors and poly(A) tail sequences. Eliminate reads with an N content greater than 5% using a Perl script. Reads with a base quality of 20 or higher were extracted, representing 70% of the total, using FASTX Toolkit software version 0.0.13. Repair the paired-end sequences using BBMap software. Finally, the fragments of the filtered high-quality reads were aligned to the mouse reference genome using the hisat2 software (version 0.7.12) [32, 33].

### RT-qPCR

Total RNA was extracted from tissues and cells using Trizol (16096020, Thermo, USA). Thermo Scientific assessed the concentration and purity of the RNA using the NanoDrop One/OneC microvolume nucleic acid and protein concentration analyzer. The A260/A280 ratio was 2.0, and the concentration exceeded 5 µg/µL. RNA was synthesized using the cDNA First Strand Synthesis Kit (D7168L, Beyotime, Shanghai).

Conduct the RT-qPCR experiment using the RT-qPCR kit (Q511-02, Vazyme Biotech, Nanjing) according to the provided instructions. Combine 2 µL of cDNA template, 0.2 µL for each of the upstream and downstream primers, and 10 µL of RT-qPCR Mix. Supplement with RNAase-free water to reach a total volume of 20 µL. Carry out the PCR amplification using the Bio-rad CFX96 real-time PCR machine with the specified reaction conditions: pre-denaturation at 95 ℃ for 30 s, denaturation at 95 ℃ for 10 s, annealing at 60 ℃ for 30 s, extension at 72 ℃ for 30 s, for a total of 40 cycles. The melting curve should range from 65 ℃ to 95 ℃. The primer sequences were designed and provided by Shanghai Bioengineering Co., Ltd. The primer sequences can be found in Table S1. The term 2^−ΔΔCt^ is used to quantify the relative change in gene expression of the target gene in the experimental group compared to the control group, with GAPDH serving as the reference gene [34]. The formula is as follows: ΔΔCt = ΔCt of the experimental group minus ΔCt of the control group, where ΔCt = Ct (target gene) minus Ct (internal reference). The experiment was repeated three times.

### Western blot

Initially, the cells underwent lysis using RIPA lysis buffer (P0013B, Beyotime, Shanghai) containing 1% PMSF to extract the total protein. Subsequently, the total protein concentration of each sample was determined using the BCA assay kit (P0011, Beyotime, Shanghai). An 8-12% SDS gel should be prepared to accommodate the target protein’s size. Then, equal amounts of protein samples should be loaded into each lane using a micropipette for electrophoresis separation. The protein was transferred onto a PVDF membrane (1620177, BIO-RAD, USA) and then blocked with 5% skim milk at room temperature for one hour. Include the following antibodies: anti-CSF1R (ab254357, 1:1000, Abcam), anti-p-STAT3 (ab76315, 1:2000, Abcam), anti-STAT3 (ab68153, 1:1000, Abcam), anti-CPT1A (ab128568, 1:1000, Abcam), anti-CPT1B (PA5-86996, 1:1000, Thermo), anti-GAPDH (ab8245, 1:2000, Abcam). Incubate overnight at 4 °C. Subsequently, wash thrice with 1× TBST washing solution at room temperature, allocating 5 min for each wash cycle. Incubate the sample with goat anti-rabbit IgG (ab6721, 1:2000) conjugated to horseradish peroxidase (HRP) or goat anti-mouse IgG (ab6728, 1:2000) conjugated to HRP for one hour at room temperature. Subsequently, the specimens were washed thrice with 1×TBST buffer at room temperature, lasting 5 min. The ECL reaction solution (1705062, Bio-Rad, USA) was added, and band exposure imaging was performed using the Image Quant LAS 4000 C gel imaging system (GE, USA) [35]. To determine the relative expression level of the protein, GAPDH was used as an internal control. The ratio of grayscale values between the target band and the internal control band was analyzed using ImageJ software (V1.8.0.112). Three replicates were performed for each experimental group.

### ELISA

ELISA assays were conducted using Mouse iNOS ELISA Kit (ab253219), Mouse Arginase 1 ELISA Kit (ab269541), Mouse TGF-beta 1 ELISA Kit (ab119557), Mouse IL-10 ELISA Kit (ab255729), Mouse IFN-beta ELISA Kit (ab252363), and Mouse Granzyme B ELISA Kit (ab238265) purchased from Abcam in the UK. The Mouse CXCR2 ELISA Kit (ABIN6954692) was obtained from antibodies online. The experiment should follow the instructions provided in the reagent kit [36].

### CD8^+^ T cell in vitro suppression assay

In the T cell-extracellular inhibition assay, activated CD8 + T cells and MDSCs were co-cultured to observe the proliferation of CD8 + T cells. Initially, CD8 + T cells were isolated from C57BL/6 mice using a mouse CD8 + T cell enrichment kit (8804-6822-74, Thermo, USA) and labeled with CellTrace Violet (C34557, Thermo, USA). They were then activated with anti-CD28 monoclonal antibody (ab243228, Abcam, UK), and MDSCs were subsequently added at different ratios to the culture. Finally, flow cytometry was used to analyze the proliferation of CD8 + T cells labeled with CellTrace Violet [37].

### Tumor vaccination and treatment

We acquired male BALB/c mice (strain code: 201) aged 3–5 weeks from Beijing Vetonic Lihua Experimental Animal Co., Ltd. To create a colon tumor model, subcutaneous implantation of 1 × 10^6^ CT26 cells (CL-0071, Wuhan Pumanase Life Sciences Co., Ltd.) was performed on the backs of mice. The formula for calculating tumor volume is expressed as volume (mm3) = length × (width)²/2. The size of the tumor is measured daily using a caliper. Once the tumor size reaches 200 mm3, the mice are randomly assigned to the following treatment groups: (1) PBS group (control group, receiving intravenous injection of equal volume of PBS); (2) LNCs@CSF1R siRNA group (receiving intravenous injection of LNCs@CSF1R siRNA at a dose of 8 mg/kg body weight per mouse every two days, for a total of 5 injections); (3) anti-PD-1 group (receiving intraperitoneal injection of 200 µg anti-PD-1 (Clone: RMP1-14, BioXcell) every other day, for a total of three injections); (4) Combined group (simultaneous treatment of mice with LNCs@CSF1R siRNA and anti-PD-1). After 28 days, the mice were euthanized using an intraperitoneal injection of sodium pentobarbital (100 mg/kg) to induce overdose anesthesia. The tumors were then excised and weighed [37].

### Extracellular flux analysis

The Seahorse XFe96 Extracellular Flux Analyzer (Agilent Technologies) was used to analyze cellular metabolism. The extracellular acidification rate (ECAR) and oxygen consumption rate (OCR) were calculated for each well. The following concentrations of injection compounds were simultaneously applied for the XF glycolysis stress or XF cell Mito test: 10 mM of glucose (CAS No.: 50-99-7, Sigma-Aldrich, USA), 2 µM of Oligomycin (CAS No.: 1404-19-9, Sigma-Aldrich, USA), 50 mM of 2-deoxy-D-glucose (2-DG) (CAS No.: 154-17-6, Sigma-Aldrich, USA), 1 µM of carbonyl cyanide 4-(trifluoromethoxy)phenylhydrazone (FCCP) (CAS No. : 370-86-5, Sigma-Aldrich, USA), and 0.5 µM of rotenone (CAS No. : 83-79-4, Sigma-Aldrich, USA). The XF sugar fermentation, stress-induced, or XF cell Mito detection assay kit was purchased from Agilent Technologies [38].

### Glucose and fatty acid intake experiment

Sugar intake was assessed through a flow cytometry-based assay. Single-cell suspensions were incubated with 100 µM of fluorescently labeled 2NBDG (ab287845, Abcam, UK) for 2 h. Subsequently, flow cytometry analysis was conducted to determine the concentration of 2NBDG within the cells.

The uptake of fatty acids in MDSCs was determined using a fluorescence fatty acid uptake assay kit (ab287857, Abcam, UK) in accordance with the instructions provided. After one hour of serum-starvation treatment at 37 °C, the cells were incubated with a mixture of fatty acids for 30 min. Subsequently, the fluorescence signal was measured using an enzyme-linked immunosorbent assay (ELISA) reader (BIO-RAD) [29].

### Cytogenetic analysis of histology

Hematoxylin and eosin staining was conducted as follows: Tumor tissues were collected from each group of mice, fixed in 10% neutral formalin, embedded in paraffin, and subsequently subjected to sectioning. The tissue slice is first dewaxed using xylene, then stained with hematoxylin, washed with distilled water, soaked in 95% ethanol, stained with eosin, dehydrated using ethanol, and finally air-dried for observation under an optical microscope [39].

Immunohistochemistry was performed on the tumor sections from each group of mice using the standard staining protocol [40]. The antibodies used in this study were Ki-67 (1:200, ab15580, Abcam, UK), CD11b (1:500, ab133357, Abcam, UK), and CSF1R (1:100, ab254357, Abcam, UK).

TUNEL staining was conducted on tumor tissues from each group of mice using the one-step TUNEL cell apoptosis detection kit (C1086, Bi Yun Tian, Shanghai). The dried slides were fixed with 4% paraformaldehyde at room temperature for 30 min, washed three times with phosphate-buffered saline (PBS), and then permeabilized with 1% Triton X-100 at 4 °C for 4 min. Each section was then transferred to a mixture of nucleotides labeled with terminal deoxynucleotidyl transferase (TdT) and incubated at 37 °C for 60 min in darkness. The samples were washed twice with PBS and stained with DAPI at room temperature for 5 min. Under a fluorescence microscope, TUNEL-positive cells exhibit green fluorescence [41].

### Statistical analysis

The research utilized R programming language, specifically version 4.2.1, in combination with the integrated development environment RStudio (version 2022.12.0-353). All data were analyzed using GraphPad Prism 8.0. The mean ± standard deviation (Mean ± SD) was used to express quantitative data. Non-paired t-tests were conducted to compare two groups, while one-way analysis of variance (ANOVA) was used for comparisons among multiple groups. The homogeneity of variance was assessed using Levene’s test. When the assumption of homogeneity of variance was met, pairwise comparisons were performed using Dunnett’s T3 and LSD-t tests. The Dunnett’s T3 test is employed when the variances are unequal. A p-value less than 0.05 indicates a statistical difference in comparison between the two groups [42].

## Results

### Single-cell sequencing analysis reveals distinct cell types in normal and colorectal Cancer tissues

Colorectal cancer is a malignancy that impacts global cancer incidence and mortality rates, particularly in developed countries and regions undergoing industrialization [43]. Although there have been recent advances in treatment methods, the management of colon cancer continues to encounter several challenges, including delayed diagnosis, recurrence, and the development of drug resistance [44].

To delve deeper into the mechanisms of colorectal cancer and explore potential therapeutic strategies, we acquired a single-cell sequencing dataset, GSE200997, specifically related to colorectal cancer, from the GEO database. The dataset encompasses 7 samples of normal colon tissue and 16 samples of colorectal cancer tissue. The data is integrated using the “Seurat” package, and normal samples are merged as “Normal”, while colon cancer tissues are merged as “Tumor”. First, we examined the number of genes (nFeature_RNA), the number of mRNA molecules (nCount_RNA), and the percentage of mitochondrial genes (percent.mt) for all cells in the dataset. The results indicated that most cells had nFeature_RNA values less than 5000, nCount_RNA values less than 20000, and percent.mt values less than 20% (Figure S3A). After removing low-quality cells that met the criteria of nFeature_RNA > 200, nFeature_RNA < 5000, and percent.mt < 10, we obtained an expression matrix with 18,749 genes and 59,431 cells remaining. The correlation analysis of sequencing depth indicates that there is a negative correlation coefficient (*r* = -0.16) between filtered nCount_RNA and percent.mt, and a positive correlation coefficient (*r* = 0.71) between filtered nCount_RNA and nFeature_RNA (Figure S3B-C). Therefore, it could be concluded that the filtered cell data is good quality and suitable for the next analysis stage.

Additional analysis was conducted on the filtered cells to select highly variable genes based on gene expression variance. The top 2000 genes with the highest variance were selected for subsequent analysis (Figure S3D). Compute the cell cycle of the samples using the CellCycleScoring function (Figure S3E), followed by initial data normalization. Afterward, Principal Component Analysis (PCA) is applied to the data for linear dimensionality reduction based on the selected highly variable genes. In this study, we present the primary heatmap illustrating the expression of genes for PC_1 to PC_6, as shown in Figure S3F. Additionally, Figure S3G visualizes the distribution of cells in PC_1 and PC_2. The findings demonstrate the existence of batch effects within the samples.

**Fig. 1:**
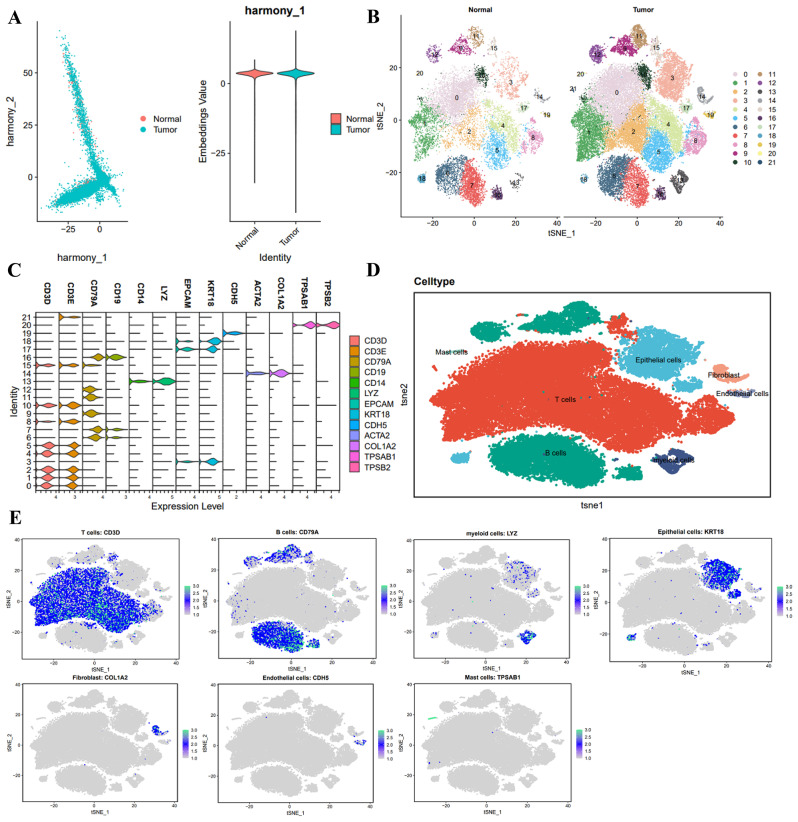
Colon cancer scRNA-seq data cell clustering. *Note* (**A**) Distribution of cells after batch correction using Harmony in PC_1 and PC_2, where each point represents a cell; (**B**) Visualization of tSNE clustering results, showing the clustering and distribution of cells in Normal and Tumor samples, with each color representing a cluster; (**C**) Expression of known cell lineage-specific marker genes in different clusters, shown as violin plots; (**D**) Visualization of cell annotation results based on tSNE clustering, with each color representing a cell subpopulation; (**E**) Distribution of marker genes for each cell type, where darker blue indicates higher average expression levels. Normal *n* = 7, Tumor *n* = 16

Batch correction of the sample data was conducted using the “harmony” package to mitigate batch effects and enhance the accuracy of cell clustering. It is illustrated in Figure S3H. Furthermore, ElbowPlot was employed to rank the principal components (PCs) based on standard deviation. The results revealed that PC_1-PC_20 effectively captured the information within the chosen highly variable genes and were analytically significant, as depicted in Figure S3I. The corrected results demonstrate the successful elimination of the batch effect from the samples, as shown in Fig. 1A.

Furthermore, the t-SNE algorithm was used to nonlinearly reduce the dimensionality of the first 20 principal components. By utilizing t-SNE clustering analysis, we successfully clustered all cells into 22 distinct cell clusters (Fig. 1B). A search was conducted in the relevant literature to obtain known marker genes specific to cell lineages. These genes were then annotated using the online website CellMarker (Fig. 1C), resulting in seven cell categories. Clusters 0, 1, 2, 4, 5, 8, 10, 15, and 21 were identified as T cells, with CD3D and CD3E as their marker genes. Clusters 3, 17, and 18 were identified as epithelial cells, with EPCAM and KRT18 as their marker genes. Clusters 6, 7, 9, 11, 12, and 16 were identified as B cells, with CD79A and CD19 as their marker genes. Cluster 13 was identified as myeloid cells, with CD14 and LYZ as its marker genes. Cluster 14 was identified as fibroblast, with ACTA2 and COL1A2 as its marker genes. Cluster 20 was identified as a mast cell, with TPSAB1 and TPSB2 as its marker genes (Fig. 1D). Furthermore, we presented the distribution of marker genes to validate the annotation results for each cell type, as shown in Fig. 1E.

In conclusion, our study utilized single-cell sequencing analysis of both normal and colon cancer tissue to annotate seven distinct cell types.

### Differential distribution and role of myeloid cells in colorectal cancer immune evasion

**Fig. 2:**
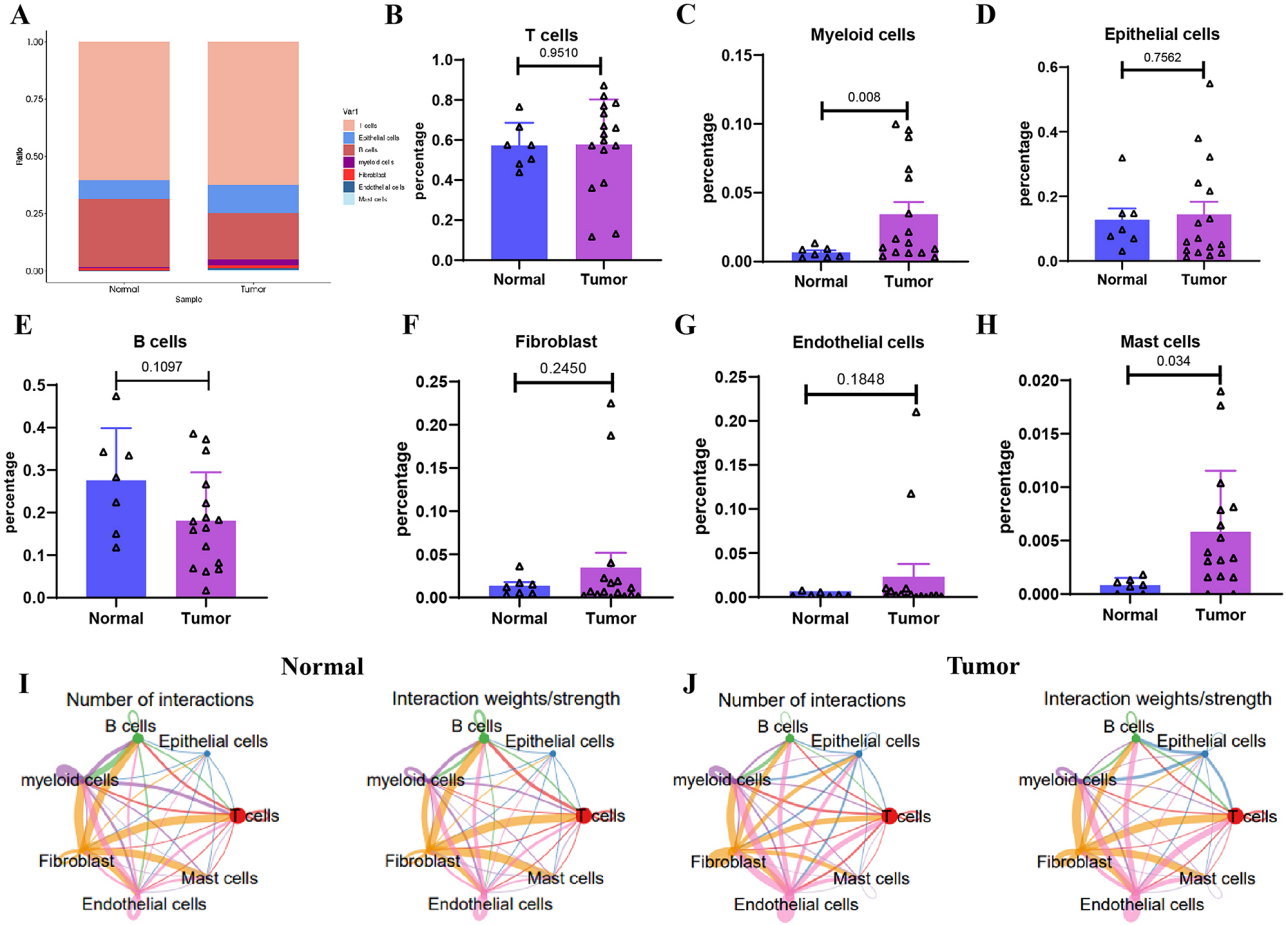
Cell numbers and cell communication analysis differ between normal colon tissues and colon cancer tissues. *Note* (**A**) Proportions of different cell subpopulations in Normal and Tumor samples, represented by different colors; (**B**-**H**) T-test analysis of cell composition differences between Normal and Tumor samples, with differences highlighted in red dashed boxes, where *P* < 0.05 indicates significance; (**I**-**J**) Circos plots of cell communication in Normal (**I**) and Tumor (**J**) samples, where the thickness of lines represents the number of pathways and the strength of interactions, respectively

Moreover, we present the distribution of cellular composition for 7 types of cells in both Normal and Tumor samples (Fig. 2A). Differences in cell quantities among different tissues were compared using t-tests. The results revealed that myeloid cells and mast cells exhibited higher proportions in colorectal cancer tissue compared to normal colon tissue, indicated by a statistical significance of (P < 0.05) (Fig. 2B-H). To better understand the underlying functional differences behind these quantitative disparities, we employed the ‘CellChat’ R package to examine the pathway activity among distinct cell types. The analysis uncovered an elevation in the interaction between Myeloid cells and T cells in the Tumor group compared to the Normal group. Conversely, the interaction between T cells and other cells weakened. Our hypothesis posits that Myeloid cells might hinder the activity of T cells through the secretion of immunosuppressive molecules, thereby reducing the interaction between T cells and other cells (Fig. 2I-J; Figure S4).

**Fig. 3:**
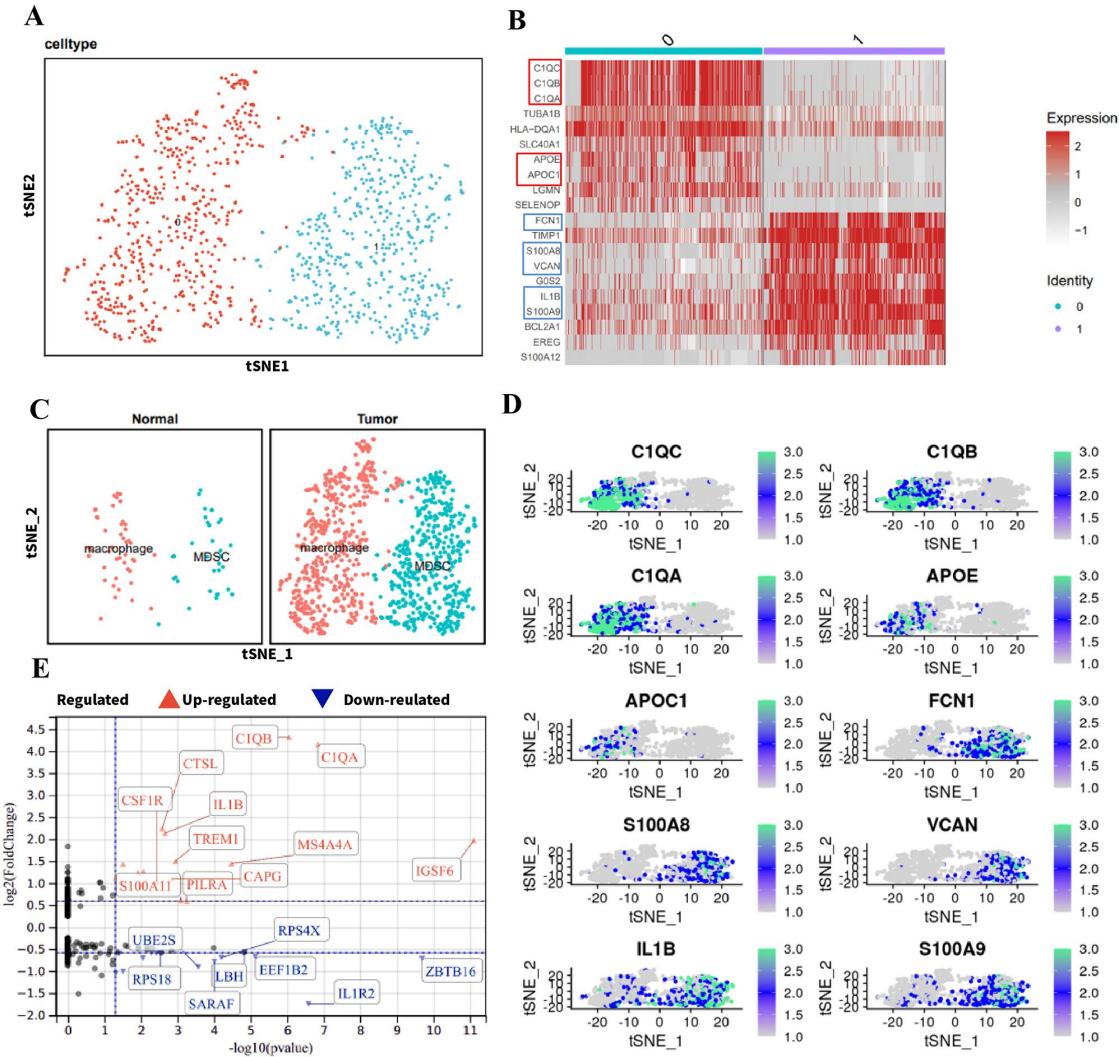
Unique gene expression characteristics of macrophages and MDSCs in Myeloid cells of Normal and Tumor groups *Note* (**A**) t-SNE analysis results showing the two main clusters of Myeloid cells extracted from Normal and Tumor groups, where each point represents an individual Myeloid cell, and similar cells cluster together in a 2D space, with cluster0 (red) and cluster1 (blue) marked with different colors; (**B**) Heatmap showing the top 10 genes with the highest expression in each cluster; (**C**) Annotation of cluster0 as macrophages and cluster1 as myeloid-derived suppressor cells (MDSCs) based on gene expression characteristics; (**D**) Expression distribution of key factors such as C1QC, C1QB, C1QA, APOE, APOC1, FCN1, S100A8, VCAN, IL1B, and S100A9 in the population of Myeloid cells; (**E**) Volcano plot showing differential genes in MDSCs between Normal and Tumor samples

Consequently, we proceeded with further extraction of the myeloid cell population in both the Normal and Tumor groups. Employing tSNE clustering, we obtained two clusters (Fig. 3A) and identified the top 10 genes in each cluster. Cluster 0 exhibited high expression of macrophage-related factors, such as C1QC, C1QB, C1QA, APOE, and APOC1, while cluster 1 showed high expression of myeloid-derived suppressor cell (MDSC)-related factors, including FCN1, S100A8, VCAN, IL1B, and S100A9 (Fig. 3B). We labeled cluster 0 as macrophages and cluster 1 as MDSCs based on these observations (Fig. 3C). The distribution of the factors above in the Myeloid cells group should also be presented, which aligns with the heatmap shown in Fig. 3D.

In colorectal cancer, myeloid-derived suppressor cells (MDSCs) exert various immunomodulatory and pro-tumor effects in the tumor microenvironment. These effects include promoting immune evasion, facilitating tumor growth, and influencing treatment responses [45]. We conducted enrichment analysis using KEGG and GO for the annotated MDSC feature genes. The results indicated that these genes were enriched in the TNF, NF-κB, and IL-17 signaling pathways (Figure S5A). Regarding the biological processes (BP) category, they were predominantly enriched in immune system processes, immune response, and defense response (Figure S5B). As for the cellular component (CC) category, the enrichment analysis showed that these genes were mainly associated with the cytoplasm, vesicle, and adherens junction (Figure S5C). In the molecular function (MF) category, the enrichment analysis revealed that these genes were primarily linked to enzyme binding, protein binding, and receptor binding (Figure S5D). Therefore, we performed additional analysis to compare the differential genes in MDSCs between Normal samples and Tumor samples, using a threshold of p.adj < 0.05 and |logFC| > 0.5. As a result, seventeen upregulated genes and twenty-six downregulated genes were identified and filtered out (Fig. 3E).

The single-cell sequencing analysis mentioned above identified distinct distributions of myeloid and mast cells between colon cancer tissues and normal tissues. Specifically, the proportion of myeloid cells increases in colon cancer tissues, and they engage in closer interaction with T cells, suggesting their vital role in immune evasion in colon cancer.

### Targeted inhibition of CSF1R in MDSCs enhances CD8 + T cell function and suppresses colorectal cancer progression in mice

**Fig. 4:**
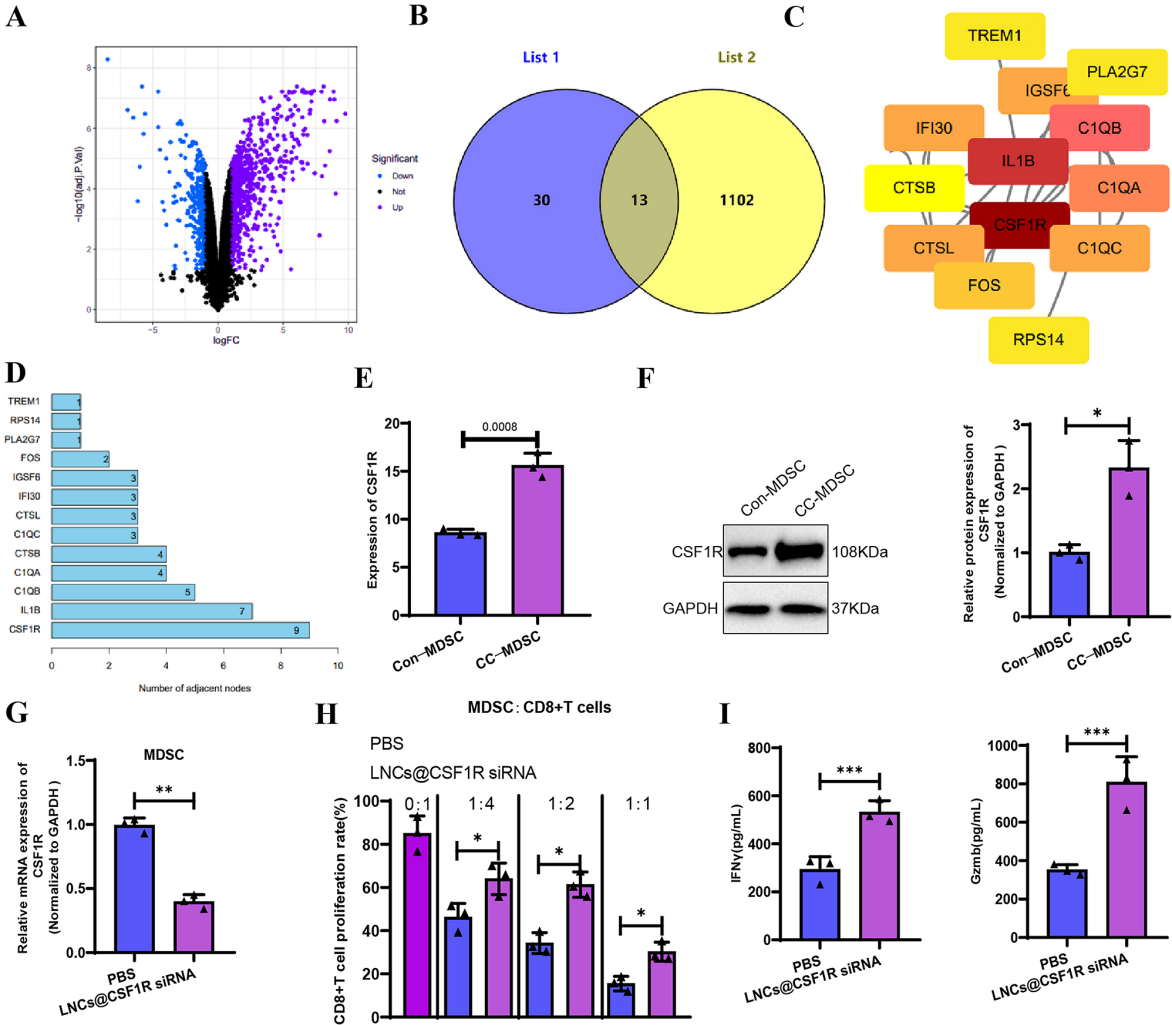
RNA-seq and in vitro experiments explore the immunosuppressive role of CSF1R in MDSCs in a colitis-associated colon cancer mouse model. *Note* (**A**) Volcano plot comparing CD11b + Gr-1 + MDSCs from tumor tissues of colon cancer mice (CC-MDSCs, *n* = 3) with BM-CD11b + Gr-1 + MDSCs from normal mice bone marrow (con-MDSCs, *n* = 3), showing 745 upregulated genes and 366 downregulated genes, with selection criteria of logFC > 1 and P-value < 0.05; (**B**) Intersection of differentially expressed genes obtained from previous scRNA-seq analysis, resulting in 13 differentially expressed genes; (**C**) Protein-protein interaction (PPI) analysis of the 13 genes; (**D**) CSF1R has the highest node degree in the PPI network; (**E**) RNA-seq results show increased expression of CSF1R in CC-MDSCs compared to con-MDSCs; (**F**) Western blot analysis confirms higher expression of CSF1R in MDSCs derived from colon cancer tissues, consistent with RNA-seq results; (**G**) qRT-PCR results show a downregulation of CSF1R transcript levels by approximately 60% in MDSCs after treatment with LNCs@CSF1R siRNA; (**H** Observation of T cell proliferation by incubating at different ratios of MDSCs to CD8 + T cells; (**I**) Detection of IFNγ and GzmB secretion in a ratio of MDSCs to CD8 + T cells of 1:1 using an ELISA kit. * represents *P* < 0.05, ** represents *P* < 0.01. Cell experiments were repeated three times

To investigate the mechanisms underlying the immunosuppressive role of MDSCs, we developed a colon cancer mouse model employing C57BL/6 mice. We isolated RNA from BM-CD11b + Gr-1 + MDSCs obtained from healthy mice (con-MDSC) and colon cancer tumor tissues acquired from mice with colon cancer (CC-MDSC) for RNA-Seq analysis. We compared the differentially expressed genes of two groups of MDSCs, where the absolute value of the logFC was greater than 1, and the P value was less than 0.05. We identified 745 upregulated genes and 366 downregulated genes (Fig. 4A). The overlap between differentially expressed genes identified from previous scRNA-seq analysis and the differentially expressed genes resulted in 13 genes (Fig. 4B). Protein-protein interaction (PPI) analysis of the proteins encoded by these 13 genes revealed that CSF1R had the highest number of interactions with other proteins (Fig. 4C) and the highest node degree (Fig. 4D).

Upon examining the RNA-seq results, we observed an upregulation of CSF1R expression, specifically in MDSCs isolated from colon cancer mouse tumor tissue (Fig. 4E). Furthermore, we conducted further investigation into the expression of CSF1R in con-MDSC and CC-MDSC using Western blot, and the obtained results agreed with RNA-seq data. CSF1R expression was higher in MDSC derived from colon cancer tissue (Fig. 4F). Furthermore, we assessed the abundance of CD11b + Gr-1 + MDSCs and the expression of CSF1R in tumor tissues at day 7, day 14, and day 28 after diagnosing colon cancer in mice. Our findings demonstrated a gradual increase in the abundance of CD11b + Gr-1 + MDSCs over time, accompanied by an elevation in CSF1R expression (Figure S6A-B). Immunohistochemical analysis of CD11b and CSF1R expression produced consistent results (Figure S6C-D).

To further explore the regulatory role of CSF1R in MDSCs in colorectal cancer, we developed a targeted MDSC-specific nanoparticle delivery system for CSF1R siRNA (LNCs@CSF1R siRNA). The procedures for preparing and characterizing the nanomaterial are documented in the Methods section, with additional details shown in Figure S1. To begin, the transcription of CSF1R was assessed following co-culturing LNCs@CSF1R siRNA with MDSCs. The results of RT-qPCR demonstrated that introducing LNCs@CSF1R siRNA suppressed CSF1R expression in MDSCs, resulting in an approximate 60% downregulation (Fig. 4G). Further examination of the impacts of various MDSC groups on CD8 + T cells indicated that the inclusion of LNCs@CSF1R siRNA considerably diminished the suppressive capacity of MDSCs on CD8 + T cell proliferation (Fig. 4H) and enhanced the secretion of IFNγ and Gzmb (Fig. 4I).

**Fig. 5:**
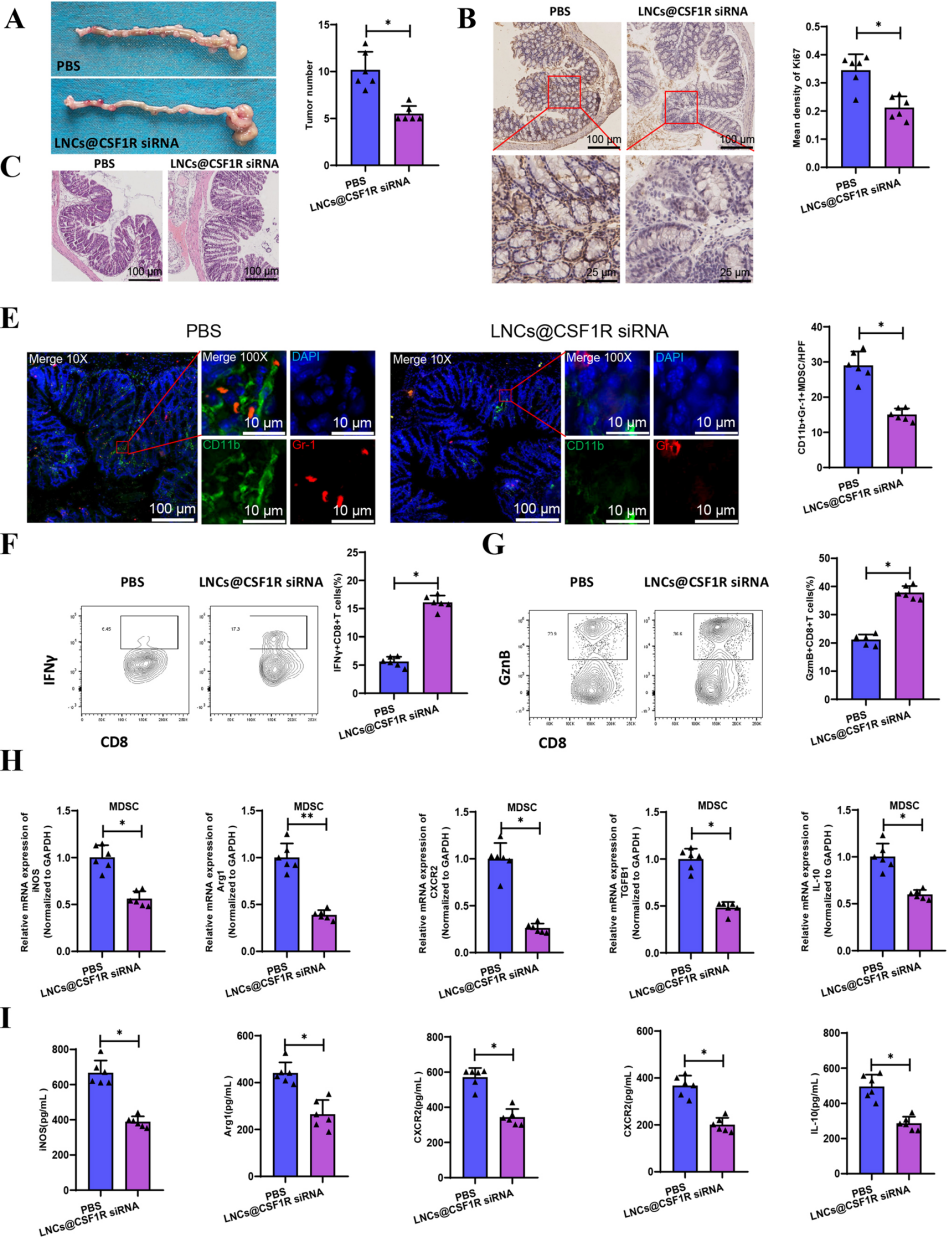
Effects of LNCs@CSF1R siRNA injection on tumor growth and immune environment in a colon cancer mouse model. *Note* (**A**) Visualization of colonic tumors in AOM and DSS-induced colon cancer mice; (**B**) Quantification of colonic tumor numbers in each group of mice; (**C**) H&E staining of tumor tissues; (**D**) Immunohistochemistry observation of the number of Ki67-positive cells; (**E**) Immunofluorescence observation of CD11b + Gr-1 + MDSCs (scale bar = 100 μm); (**F**-**G**) Flow cytometry analysis of effector cytokines IFNγ and GzmB produced by CD8 + T cells in tumor tissues after injection of LNCs@CSF1R siRNA; (**H**-**I**) RT-qPCR and ELISA analysis of mRNA and protein levels of iNOS, Arg1, CXCR2, TGF-β, and IL-10 in MDSC cells after injection of LNCs@CSF1R siRNA. * represents *P* < 0.05, ** represents *P* < 0.01. Each group consisted of 6 mice

Subsequently, LNCs@CSF1R siRNA was intravenously injected into mice with AOM- and DSS-induced colon cancer. Following injection, the large intestine of each group of mice was isolated. Our results revealed a reduction in colon tumors after injecting LNCs@CSF1R siRNA (Fig. 5A-C). Moreover, there was a decrease in the number of Ki67-positive cells within the tumors (Fig. 5D). Furthermore, by observing the changes in CD11b + Gr-1 + MDSC through immunofluorescence staining, it was found that the injection of LNCs@CSF1R siRNA decreased the presence of CD11b + Gr-1 + MDSC (Fig. 5E). Additionally, we also observed a decrease in the total formation of CD11b + Gr1 + MDSC, Ly6G + CD11b + polymorphonuclear MDSC (PMN-MDSC), and Ly6C + CD11b + monocytic MDSC (M-MDSC) using flow cytometry, with a relatively larger reduction in PMN-MDSC (Figure S7A-C). Furthermore, the injection of LNCs@CSF1R siRNA increased the production of effector cytokines IFNγ and GzmB by CD8 + T cells within the tumor (Fig. 5F-G). MDSCs were isolated from the tumor tissues of each mouse group, and the expression of immune suppressive molecules in MDSCs from each group was assessed using RT-qPCR. The results revealed a reduction in mRNA levels of iNOS, Arg1, CXCR2, TGF-β, and IL-10 in MDSCs following the injection of LNCs@CSF1R siRNA (Fig. 5H). The ELISA results also demonstrated a noteworthy reduction in the protein levels of the factors above (Fig. 5I).

Our experiments revealed that inhibiting CSF1R in MDSCs could effectively suppress their immunosuppressive effects and enhance the effector function of CD8^+^ T cells.

### Inhibition of CSF1R disrupts fatty acid metabolism in MDSCs, reducing their immunosuppressive capacity without compensatory glycolysis

**Fig. 6:**
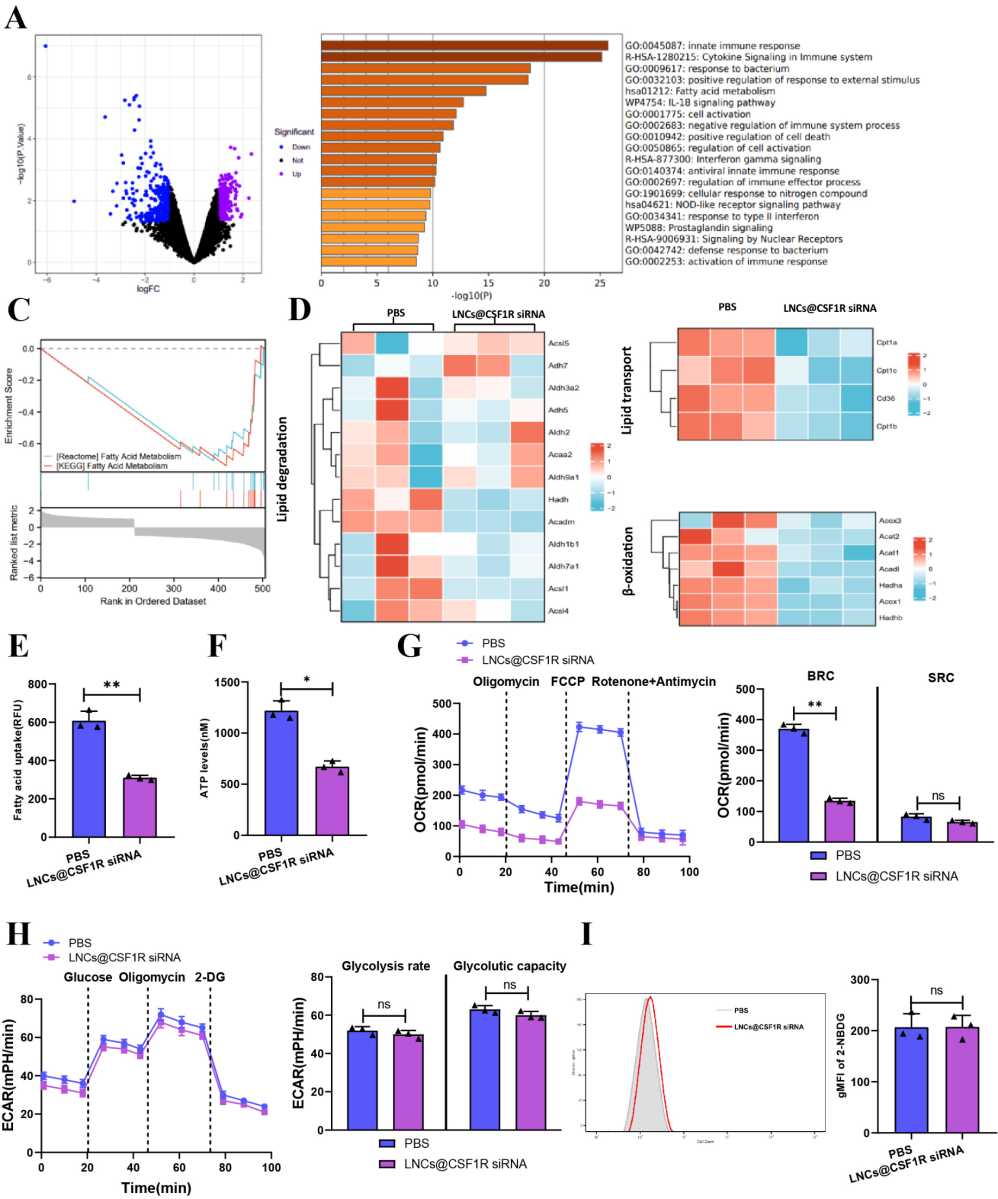
Effect of LNCs@CSF1R siRNA on gene expression and metabolic pathways in MDSCs. *Note* (**A**) Volcano plot of differentially expressed genes in MDSCs after injection of LNCs@CSF1R siRNA compared to MDSCs without LNCs@CSF1R siRNA injection; (**B**) KEGG and GO enrichment analysis of differentially expressed genes; (**C**) GSEA enrichment analysis of differentially expressed genes; (**D**) Heatmap showing differences in the expression of factors related to fatty acid degradation, transport, and beta-oxidation in MDSCs after injection of LNCs@CSF1R siRNA based on RNA-seq data; (**E**) Fluorescence fatty acid uptake assay measuring fatty acid uptake in MDSCs; (**F**) ATP levels quantified using a bioluminescence assay; (**G**) Basal respiratory capacity (BRC) and spare respiratory capacity (SRC) of MDSCs in PBS group and LNCs@CSF1R siRNA group measured using Seahorse extracellular flux analysis; (**H**) ECAR assay measuring glycolytic pathway in MDSCs in PBS group and LNCs@CSF1R siRNA group; (**I**) Flow cytometry analysis measuring uptake of 2-NBDG, a fluorescent glucose analog, in MDSCs in PBS group and LNCs@CSF1R siRNA group. * indicates *P* < 0.05, ** indicates *P* < 0.01. Cell experiments were repeated three times

To further investigate the immunosuppressive role of CSF1R on MDSC, MDSCs were isolated from tumor tissues of colon cancer mice that were injected with LNCs@CSF1R siRNA. RNA-seq analysis was performed to identify differentially expressed genes between the injected and non-injected groups of MDSCs. We used a screening criteria of logFC > 1 and pvalue < 0.05. Two hundred forty-eight upregulated and 301 downregulated genes were identified (Fig. 6A). Furthermore, previous research has demonstrated that inhibition of CSF1R could effectively suppress the immunosuppressive functions of MDSCs. By conducting RNA-seq analysis, we identified downregulation of certain immunosuppressive factors, such as Arg1, Tgfb, and Cd274, following the administration of LNCs@CSF1R siRNA (Figure S8). Further analysis of the differentially expressed genes in MDSCs after LNCs@CSF1R siRNA treatment revealed their prominent enrichment in the fatty acid metabolism pathway, as indicated in Fig. 6B-C. Thus, we illustrated the genes associated with fatty acid degradation, fatty acid transport, and fatty acid β-oxidation in RNA-seq, as shown in Fig. 6D. The results demonstrated that with the addition of LNCs@CSF1R siRNA, several factors in the MDSCs had differences. These factors include the genes related to fatty acid degradation (Acsl1, Acadm, aldh1b1, and Aldh7a1), fatty acid transport (Cpt1a, Cpt1b, Cpt1c, and Cd36), and fatty acid β-oxidation (Acox1, Acox2, and Acat1).

In our study, we conducted additional in vitro experiments to explore the influence of CSF1R on fatty acid metabolism in MDSCs. The results showed an inhibition in fatty acid uptake (Fig. 6E) and ATP production (Fig. 6F) upon adding LNCs@CSF1R siRNA. We measured the OCR using the Seahorse extracellular flux analyzer and observed that the baseline respiratory capacity (BRC) of MDSCs in the PBS group was higher than that of MDSCs in the LNCs@CSF1R siRNA group. However, the two groups had no difference in spare respiratory capacity (SRC) (Fig. 6G). Moreover, as assessed by ECAR and glucose uptake, the glycolytic pathway is unaffected by the downregulation of CSF1R. It suggests that the inhibition of CSF1R reduces MDSC fatty acid metabolism, which cannot be offset by the glycolytic pathway (Fig. 6H-I).

Based on the results above, our findings demonstrate that targeted inhibition of CSF1R impacts the fatty acid metabolism of MDSCs, specifically affecting fatty acid uptake and ATP production. Importantly, these alterations are not counterbalanced by enhanced glycolysis, highlighting the critical role of CSF1R in regulating the immunosuppressive capacity and fatty acid metabolism of MDSCs.

### CSF1R regulates MDSC fatty acid metabolism and immunosuppression through the JAK/STAT3 signaling pathway

**Fig. 7:**
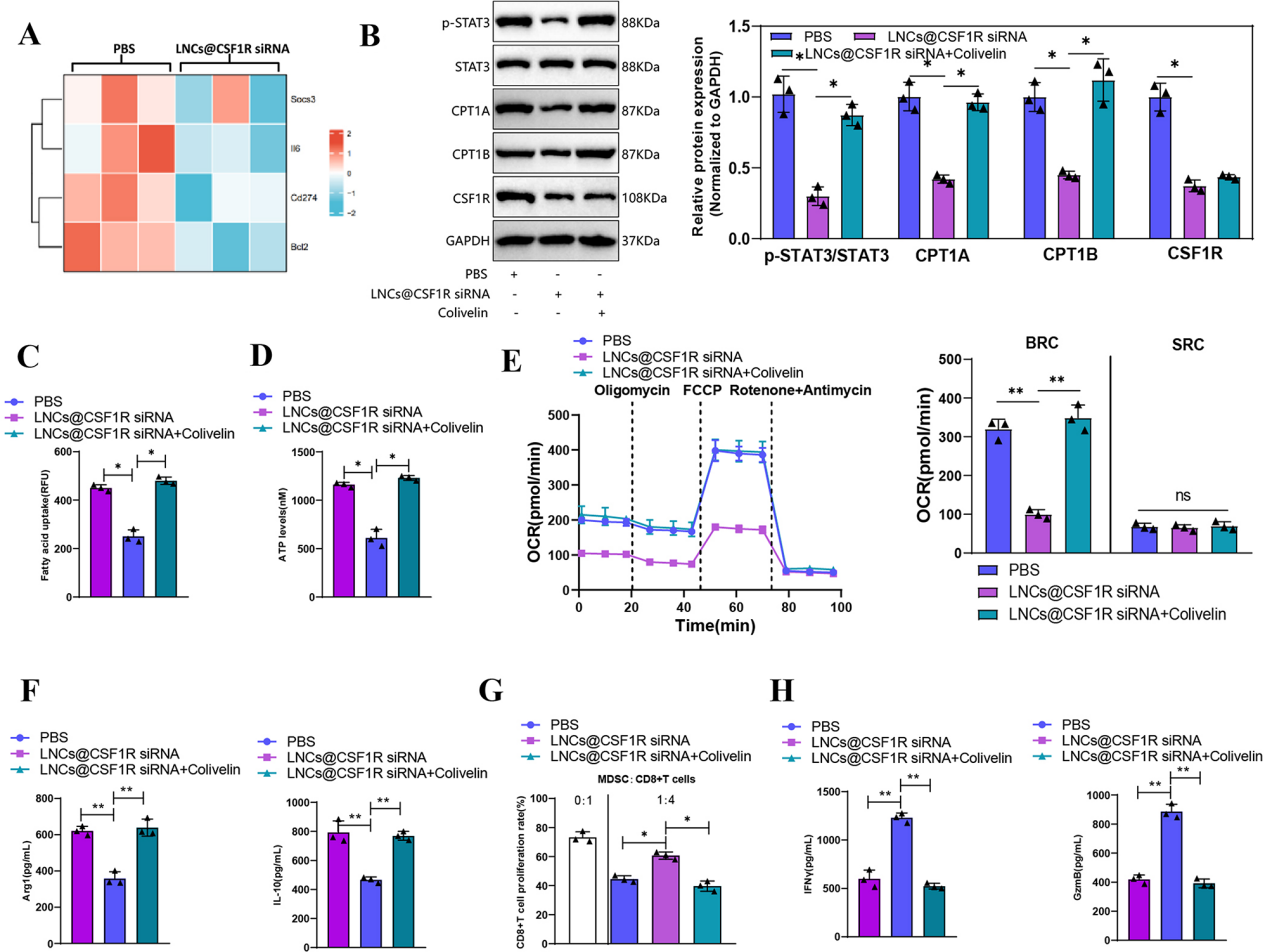
Regulation of MDSC fatty acid metabolism and immunosuppressive function by CSF1R through the JAK/STAT3 signaling axis. *Note* (**A**) Heatmap showing the expression levels of known STAT3 target genes (Cd274, Socs3, Bcl2, and Il6) in MDSCs after inhibition of CSF1R, based on RNA-seq data; (**B**) Western blot analysis showing the expression levels of p-STAT3, CPT1A, and CPT1B in MDSCs after treatment with Colivelin (a STAT3 activator); (**C**-**E**) Metabolic tests measuring fatty acid uptake, ATP generation, and respiratory capacity in MDSCs after Colivelin treatment; (**F**) ELISA measurement of Arg1 and IL-10 secretion by MDSCs after Colivelin treatment; (**G**-**H**) T cell proliferation assay and ELISA measuring the ability of Colivelin treatment to inhibit T cell proliferation and release IFNγ and GzmB. * indicates *P* < 0.05, ** indicates *P* < 0.01. Cell experiments were repeated three times

According to literature reports, CSF1R can regulate the JAK/STAT3 signaling pathway [46, 47]. Although JAK/STAT3 promotes fatty acid metabolism, it enhances it by regulating CPT1B transcription and driving CPT1A to promote fatty acid oxidation [48, 49]. To explore the potential role of CSF1R in regulating MDSC’s fatty acid metabolism through the JAK/STAT3 signaling pathway, we examined the RNA-seq data. The results demonstrated that the expression of established STAT3 target genes, including Cd274, Socs3, Bcl2, and Il6, was reduced upon CSF1R inhibition (Fig. 7A).

Colivelin, a STAT3 activator, was added to MDSC to investigate its potential to facilitate the aberrant fatty acid metabolism resulting from CSF1R inhibition. The findings indicated that the utilization of Colivelin, as a STAT3 activator, was effective in rescuing this inhibitory effect, reinstating the expression of p-STAT3, CPT1A, and CPT1B (Fig. 7B), while also enhancing fatty acid uptake, ATP release, and BCR increase (Fig. 7C-E). Furthermore, treatment with Colivelin also increased the secretion of Arg1 and IL-10 (Fig. 7F) and restored the ability of MDSCs to inhibit the proliferation of CD8 + T cells and release IFNγ and GzmB (Fig. 7G-H).

These findings confirm the crucial role of CSF1R in the fatty acid metabolism and immune suppressive function of MDSCs via the JAK/STAT3 signaling pathway.

### Combination of LNCs@CSF1R siRNA and Anti-PD-1 therapy amplifies therapeutic efficacy against colon cancer in mice

**Fig. 8:**
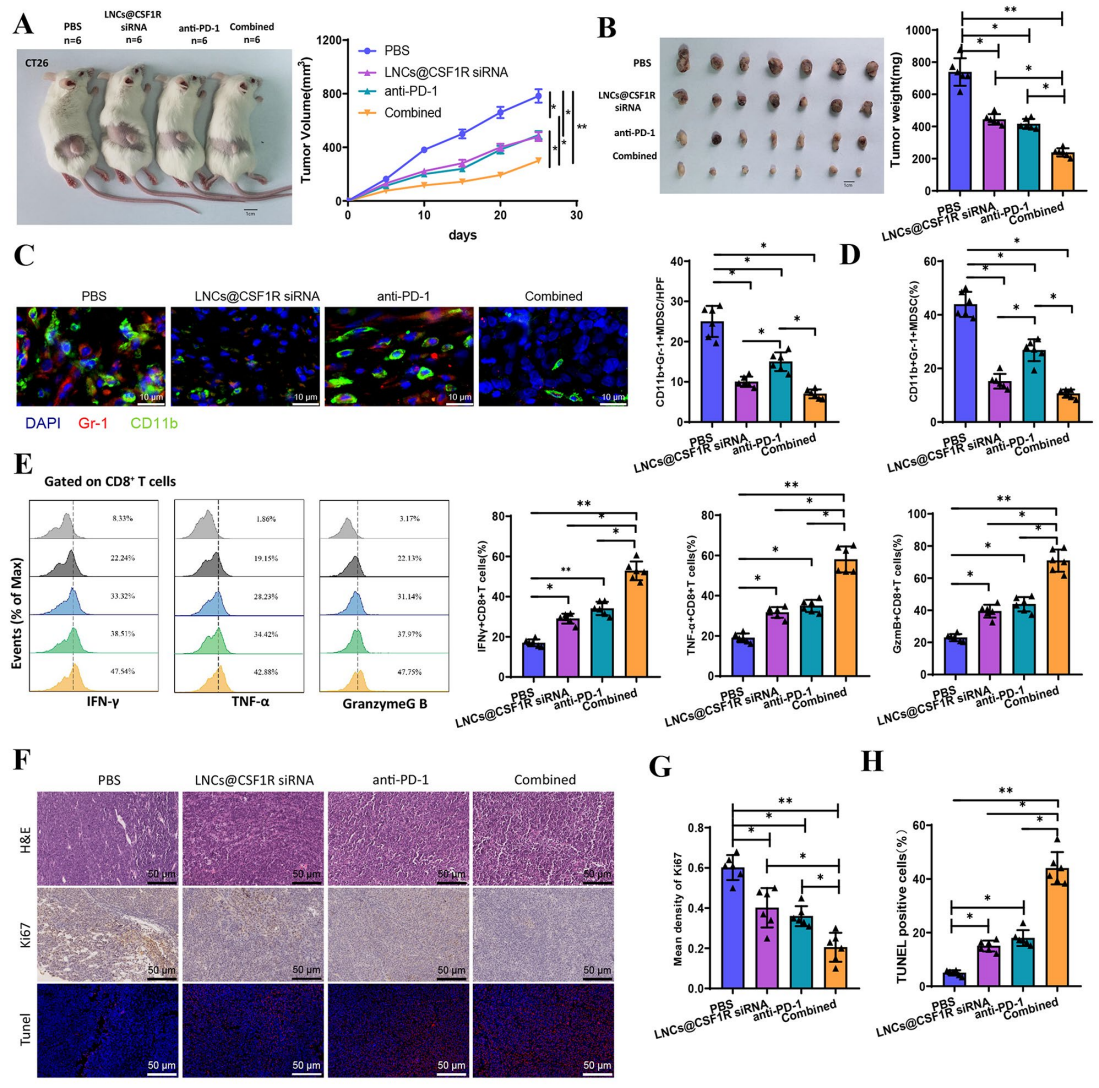
Effects of LNCs@CSF1R siRNA and anti-PD-1 monotherapy and combination therapy on colon cancer mice. *Note* (**A**) Tumor growth curves of colon cancer mice in each treatment group; (**B**) Tumor volume and mass of colon cancer mice after 28 days of treatment; (**C**) Immunofluorescence analysis of the percentage of CD11b + Gr-1 + MDSCs in tumor tissues of each group; (**D**) Flow cytometry analysis of the abundance of MDSCs in tumor tissues of colon cancer mice; (**E**) Flow cytometry analysis of the percentages of INFγ + CD8 + T cells, TNF-α + CD8 + T cells, and GzmB + CD8 + T cells in tumor tissues of colon cancer mice; (**F**-**H**) Apoptosis and proliferation rates of tumor tissues in each group assessed by H&E, Ki67, and TUNEL staining. * indicates *P* < 0.05, ** indicates *P* < 0.01. *n* = 6

Previous research has convincingly demonstrated that LNCs@CSF1R siRNA could modulate fatty acid metabolism by targeting MDSCs, which effectively inhibits their immunosuppressive effects on CD8 + T cells. This study aims to investigate the potential of LNCs@CSF1R siRNA to enhance the efficacy of anti-PD-1 therapy. A colon cancer tumor-bearing mouse model was created by subcutaneously injecting CT26 cells into BALB/c mice. Subsequently, we employed LNCs@CSF1R siRNA and anti-PD-1 combination therapy to assess the improvement of the therapeutic effect. Our findings demonstrate that individual treatments effectively inhibited tumor growth in mice with colon cancer. We observed reductions in tumor volume and weight after 28 days. Furthermore, combination therapy enhanced the effects of the individual treatments, as illustrated in Fig. 8A-B.

Additionally, we used immunofluorescence detection to analyze the proportion of CD11b + Gr-1 + MDSC in the tumors of each group of mice. The results showed decreased CD11b + Gr-1 + MDSC with both individual treatment and combination therapy. Treatment with LNCs@CSF1R siRNA alone or in combination exhibited superior inhibitory effects on CD11b + Gr-1 + MDSC compared to treatment with anti-PD-1 alone, suggesting that LNCs@CSF1R siRNA has a more effective targeting effect on CD11b + Gr-1 + MDSC (Fig. 8C). Furthermore, this result was further validated using flow cytometry (Fig. 8D; Figure S7C). Within CD8^+^ T cells, we discovered that monotherapy alone could augment the percentage of INFγ + CD8 + T cells, TNF-α + CD8^+^ T cells, and GzmB + CD8 + T cells. The combined treatment substantially intensified this outcome (Fig. 8E).

Furthermore, to assess the impact of individual and combined therapies on tumor proliferation, apoptosis, and necrosis, tumor sections from each treatment group were stained with hematoxylin and eosin (H&E), Ki67, and terminal deoxynucleotidyl transferase dUTP nick end labeling (TUNEL) as indicated in Fig. 8F-H. The combination therapy group showed the highest rate of apoptosis and the lowest rate of proliferation in the tumor tissue despite individual therapies effectively inhibiting tumor growth. The simultaneous action of LNCs@CSF1R siRNA and anti-PD-1 further enhanced this augmented therapeutic effect on mice with colon cancer.

The data presented collectively suggest that the concurrent administration of LNCs@CSF1R siRNA and anti-PD-1 could enhance the therapeutic efficacy of colon cancer in mice, exhibiting potential clinical applicability.

## Discussion

Colorectal cancer consistently ranks as one of the most frequently diagnosed malignancies worldwide, presenting significant challenges to both patients and medical professionals [2, 3, 50]. Over recent years, a growing interest has been directed toward understanding the multifaceted tumor microenvironment (TME) and its impact on therapeutic strategies.

In the midst of these research endeavors, the current study shed new light on the prominent role of CSF1R in modulating the functionality of myeloid-derived suppressor cells (MDSCs) within the TME. Notably, our research unveiled a deeper dimension to CSF1R’s function, extending beyond its previously characterized influence on the proliferation and differentiation of myeloid cells [51]. We identified that CSF1R plays a pivotal part in the fatty acid metabolism of MDSCs, which further impacts their immune-suppressive capabilities. This revelation positions CSF1R as a key player in the broader cancer biology landscape and underscores its potential as a therapeutic target.

In our pursuit of effective therapeutic modulation, we harnessed innovative nanotechnology to engineer LNCs@CSF1R siRNA nanocarriers. This novel construct was shown to possess an impressive specificity for MDSCs, representing a marked improvement over earlier interference technologies [52]. As such, the nanocarrier not only serves as an indispensable tool for probing CSF1R functions but also heralds a promising future in cancer treatment regimens.

Our investigations delved deeper into the interplay between CSF1R and the JAK/STAT3 signaling pathway, unraveling their combined effect on MDSCs. While prior research efforts predominantly concentrated on JAK/STAT3’s role in cellular processes like differentiation and proliferation, our study brought forth its hitherto uncharted regulatory role in colon cancer [53]. This fresh insight augments our understanding of the elaborate mechanisms underlying tumor immune evasion.

Validating these cellular and molecular insights, our experiments in a mouse model of colon cancer elucidated the tangible therapeutic benefits of a combined regimen of LNCs@CSF1R siRNA nanocarriers and anti-PD-1 antibodies. Compared to traditional monotherapies, our combinatorial approach manifested superior efficacy in curbing tumor growth.

Yet, while our findings significantly advance the field, it’s vital to approach them with circumspection. The study’s primary focus on murine models necessitates caution in translating these outcomes directly to human clinical scenarios. Moreover, while our combination therapeutic strategy displayed profound potential in mice, its long-term implications, safety profile, and other unforeseen effects require rigorous evaluation in subsequent research.

**Fig. 9:**
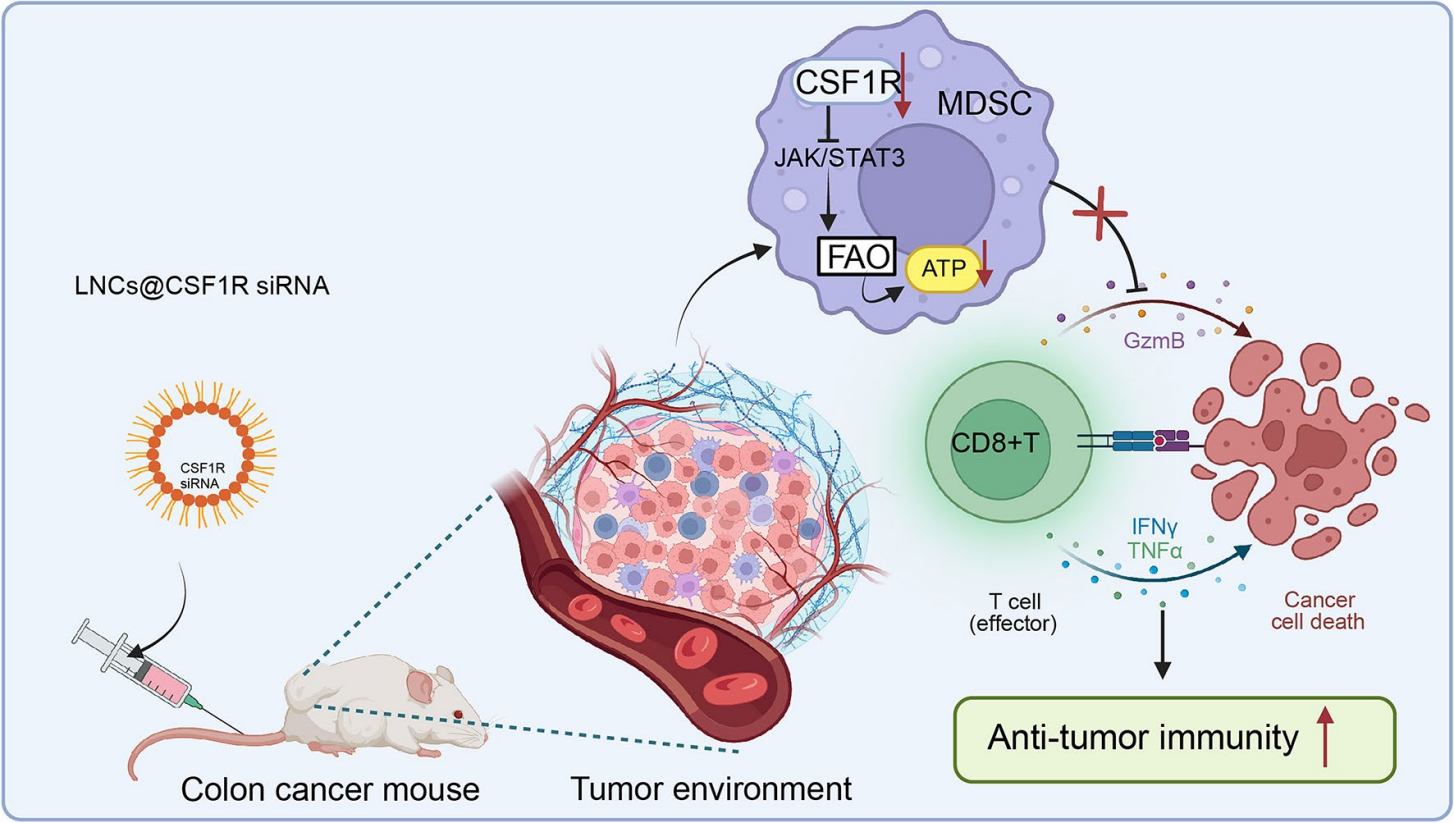
Targeting CSF1R regulates MDSC fatty acid metabolism, attenuates its immunosuppressive effect on CD8 + T cells, and promotes colon cancer immunotherapy

In conclusion, our study has woven together intricate cellular mechanisms, innovative therapeutic strategies, and pre-clinical validations, offering a comprehensive narrative on the potential avenues for colorectal cancer treatment (Fig. 9). However, the journey from bench to bedside is intricate and demands continued investigation, not only into the combined therapeutic potential of LNCs@CSF1R siRNA and anti-PD-1 but also into the broader implications of MDSC regulation in various cancer types. As we forge ahead, integrating these findings with existing treatments, such as chemotherapy or radiation, may unveil holistic and more effective treatment paradigms.

### Electronic supplementary material

Below is the link to the electronic supplementary material.

Supplementary Material 1

Supplementary Material 2

Supplementary Material 3

Supplementary Material 4

Supplementary Material 5

Supplementary Material 6

Supplementary Material 7

Supplementary Material 8
