# Supplementary figures and images for: Targeting CSF1R in myeloid-derived suppressor cells: insights into its immunomodulatory functions in colorectal cancer and therapeutic implications
Source: J Nanobiotechnology. 2024 Jul 11;22:409. doi: 10.1186/s12951-024-02584-4 (PMC11238447; doi:10.1186/s12951-024-02584-4)

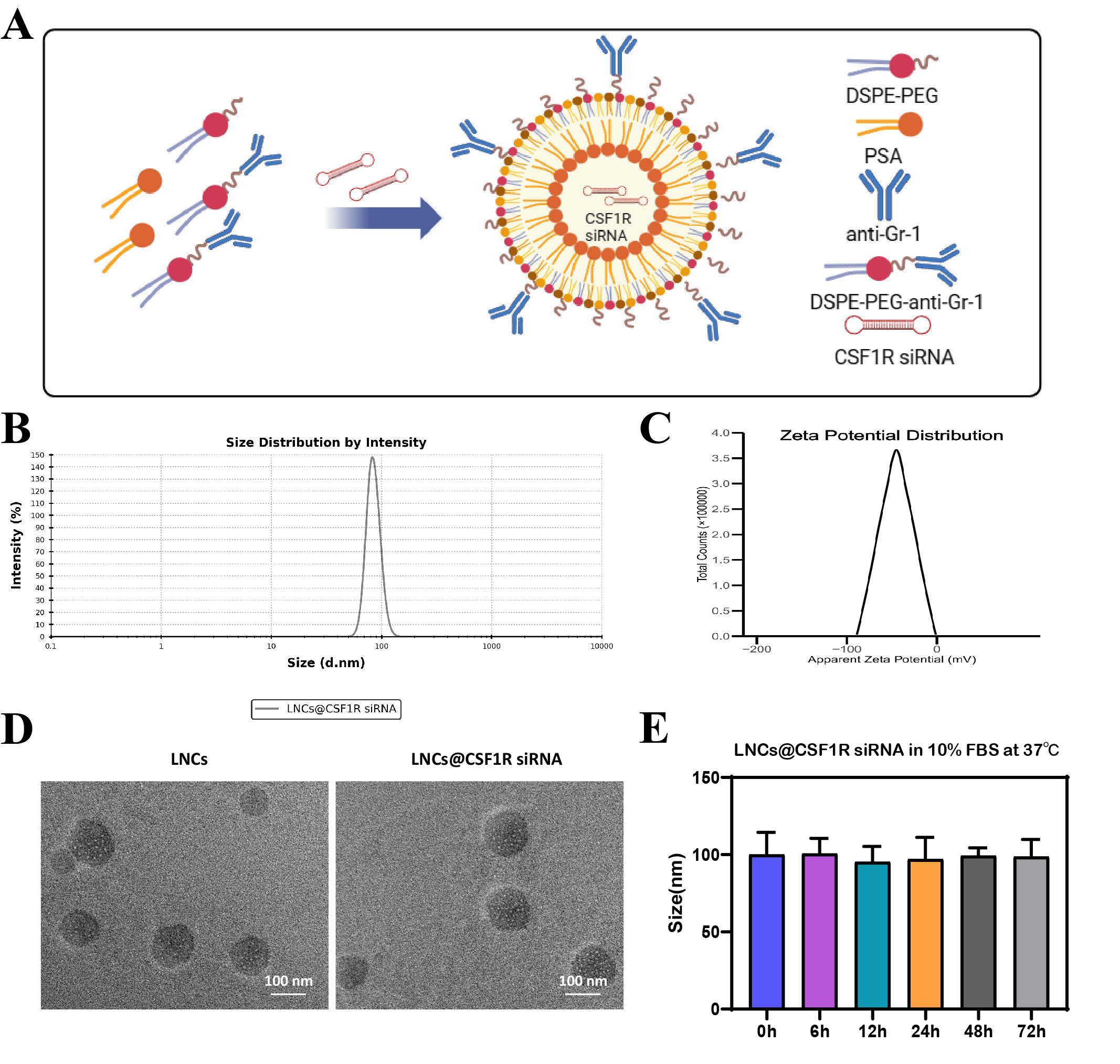

Supplement: Supplementary file 1 — Supplementary Material 1 [file 12951_2024_2584_MOESM1_ESM.jpg]

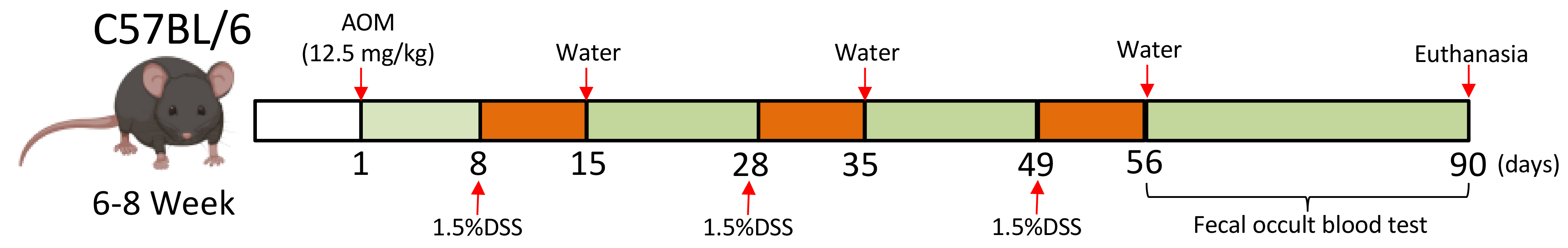

Supplement: Supplementary file 2 — Supplementary Material 2 [file 12951_2024_2584_MOESM2_ESM.jpg]

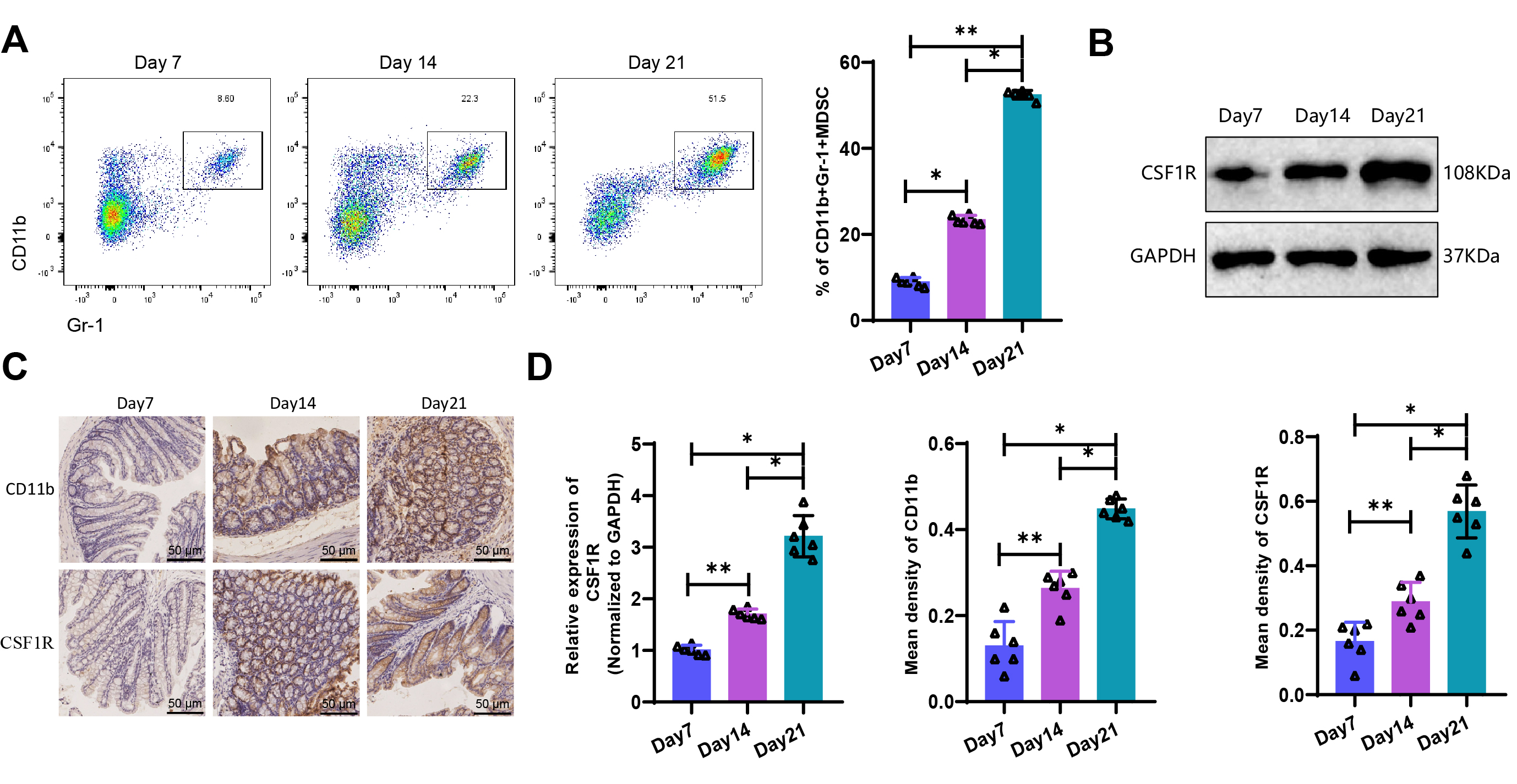

Supplement: Supplementary file 3 — Supplementary Material 3 [file 12951_2024_2584_MOESM3_ESM.jpg]

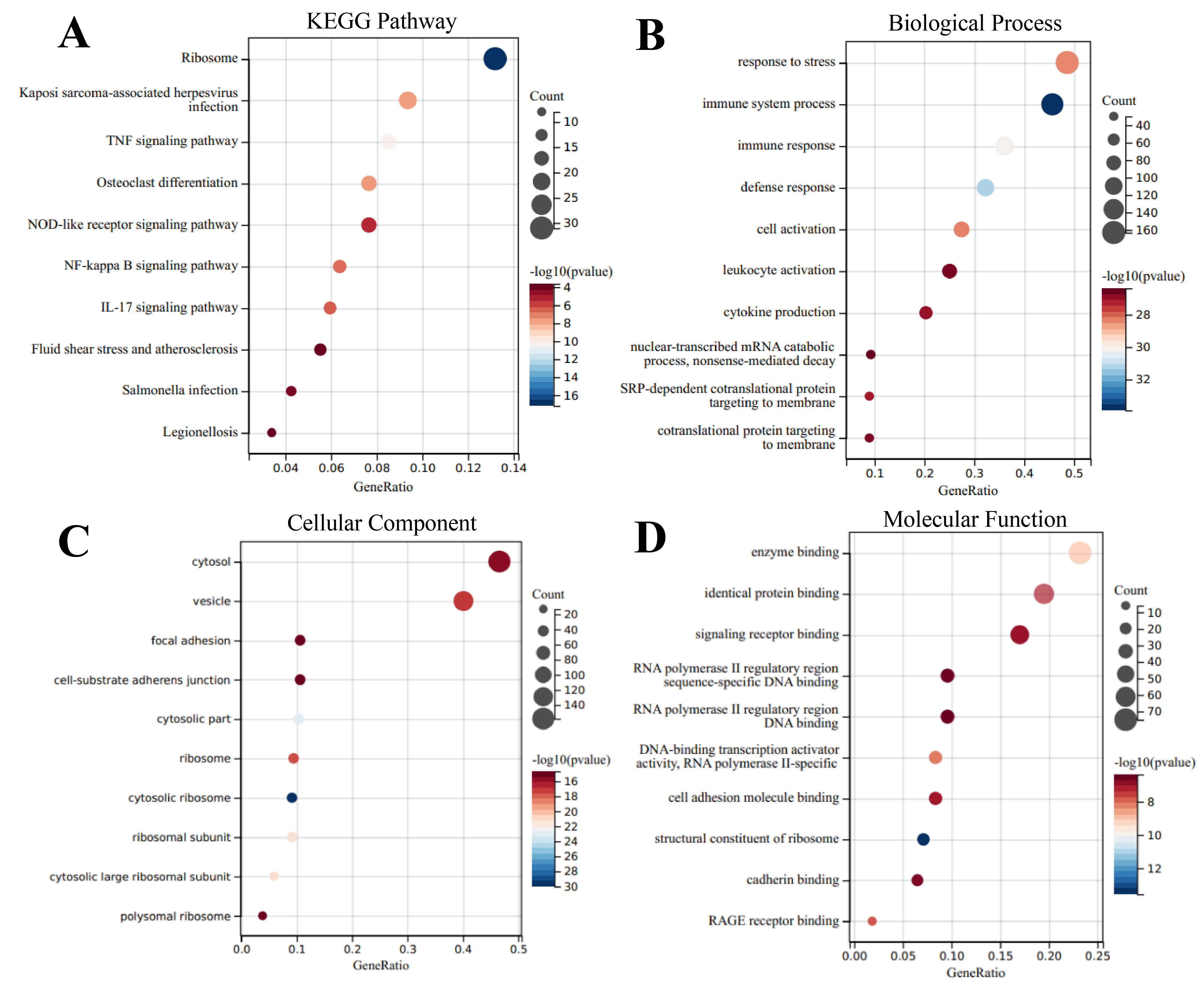

Supplement: Supplementary file 4 — Supplementary Material 4 [file 12951_2024_2584_MOESM4_ESM.jpg]

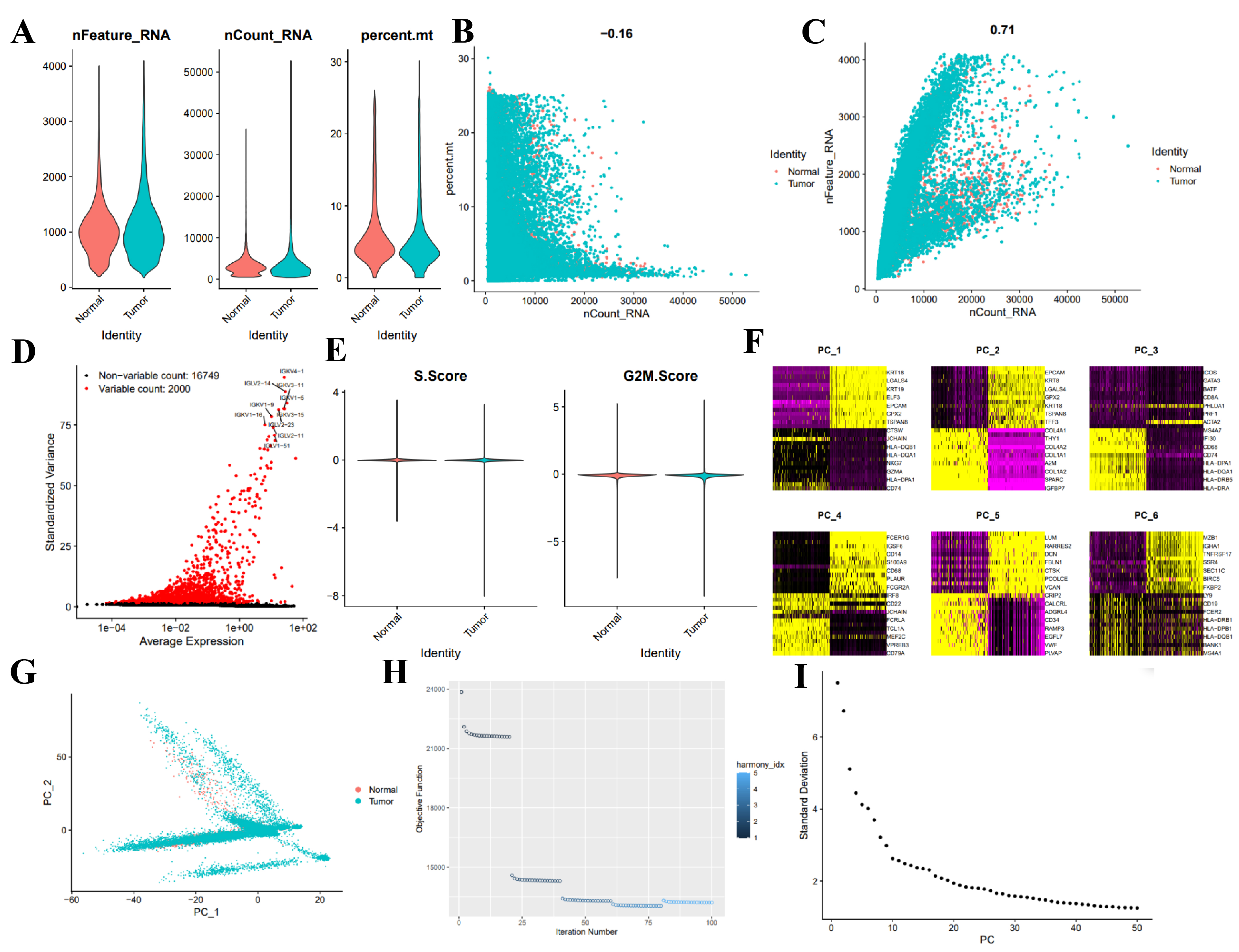

Supplement: Supplementary file 5 — Supplementary Material 5 [file 12951_2024_2584_MOESM5_ESM.jpg]

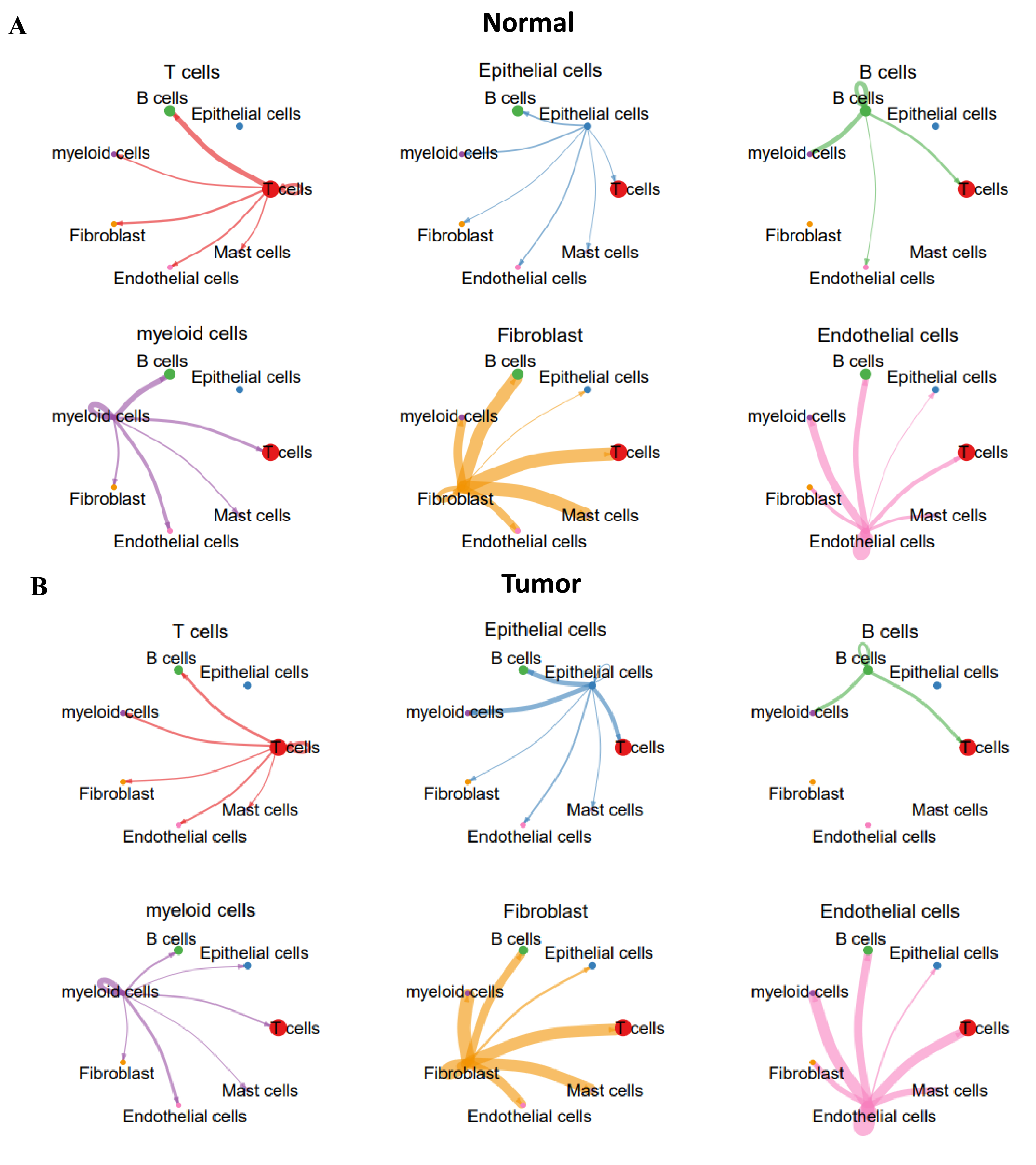

Supplement: Supplementary file 6 — Supplementary Material 6 [file 12951_2024_2584_MOESM6_ESM.jpg]

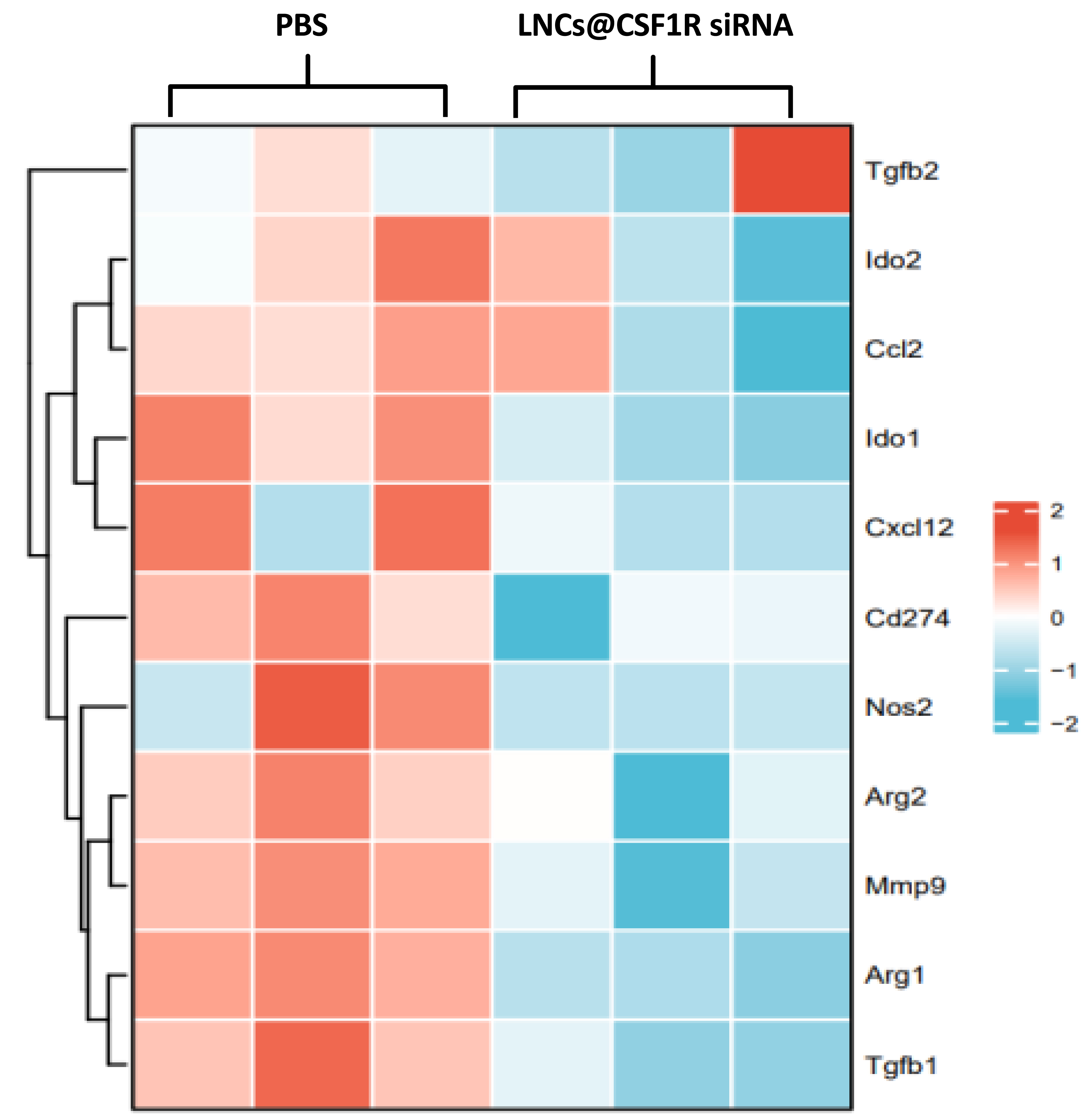

Supplement: Supplementary file 7 — Supplementary Material 7 [file 12951_2024_2584_MOESM7_ESM.jpg]

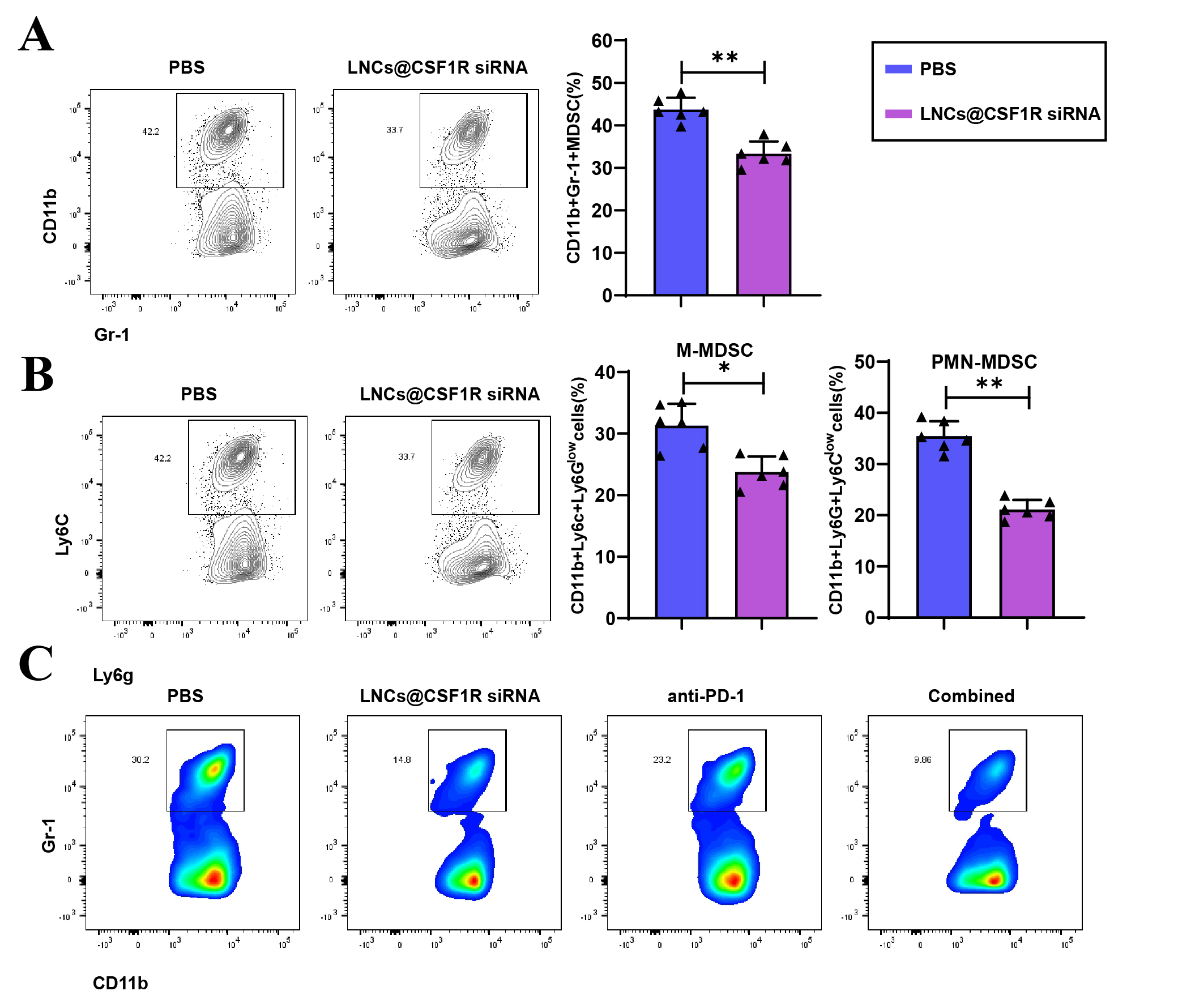

Supplement: Supplementary file 8 — Supplementary Material 8 [file 12951_2024_2584_MOESM8_ESM.jpg]
